# Economic evaluations of RSV preventive strategies: a systematic review of cost-effectiveness and modeling approaches

**DOI:** 10.3389/fpubh.2025.1672683

**Published:** 2025-10-13

**Authors:** Bingde Zhu, Yuqiong Lu, Yang Zhou, Weixiao Li, Yuhang Wu, Yujin Bao, Yun Lu

**Affiliations:** School of International Pharmaceutical Business, China Pharmaceutical University, Nanjing, China

**Keywords:** economic evaluation, cost-effectiveness, respiratory syncytial virus vaccines, systematic review, monoclonal Abs

## Abstract

**Background:**

Respiratory syncytial virus (RSV) causes significant morbidity and mortality worldwide, particularly in high-risk groups. Despite the availability of preventive interventions, it is crucial to evaluate the economic benefits of these interventions.

**Methods:**

This systematic review assessed the cost-effectiveness and model structures of RSV prevention strategies, including vaccines and monoclonal antibodies, by analyzing studies published up to March 2025.

**Results:**

A total of 39 studies were included, comprising one cost–benefit analysis (CBA) and 38 cost–effectiveness analyses (CEAs), utilizing six different types of economic models. The incremental cost-effectiveness ratio (ICER) among the older adult population varied from $5,342 to $385,829 per quality-adjusted life year (QALY). One study demonstrated superior cost-effectiveness of a long-acting monoclonal antibody (LAMA) compared to a short-acting monoclonal antibody, with both being more economically favorable than maternal vaccines for pregnant women and neonates. The most sensitive variables were intervention efficacy, price, and immunity duration.

**Conclusion:**

Most RSV vaccines and monoclonal antibody interventions demonstrate cost-effectiveness in specific populations and settings. However, cost-effectiveness is highly influenced by intervention price, efficacy, duration, populations, and administration time.

**Systematic review registration:**

The protocol for this study has been registered with PROSPERO under the registration number CRD42024524720.

## 1 Introduction

Respiratory syncytial virus (RSV) is a significant viral pathogen causing respiratory infections in infants, the older adult, and immunocompromised individuals, resulting in a substantial disease burden worldwide annually (1). RSV is a leading cause of acute lower respiratory tract infections. In severe cases, it can lead to fatal complications or repeated infections throughout life (2, 3). Recent data from 2019 indicate that around 33 million children under five worldwide were affected by acute lower respiratory tract infections due to RSV, resulting in 3.6 million hospitalizations and 26,300 in-hospital deaths (4, 5). Given that the majority of RSV-related fatalities occur outside hospital settings, the actual burden is likely underreported (6, 7). RSV infection can place a substantial economic burden on healthcare systems, especially during the peak RSV season in temperate regions (8, 9). The total global healthcare expenditure for children under 5 years old was approximately US$5 billion in 2017 (10).

Currently, there are no specific antiviral therapies available for the treatment of RSV infection, and post-infection management primarily relies on supportive care (11). To mitigate the impact of RSV, various preventive measures have been developed and are being implemented worldwide. Three vaccines and two monoclonal antibody (mAb) interventions have been approved for marketing, and dozens of small-molecule inhibitors are in clinical trials (12). Palizumab was the first commercial humanized monoclonal antibody against RSV, which was approved in 1998 for the prevention of RSV infection in infants (13). For the same population, nirsevimab, a long-acting monoclonal antibody (LAMA), received FDA approval in June 2023 (14). RSV vaccines, Arexvy, ABRYSVO, and mRESVIA have been approved by FDA in May 2023, June 2023, and May 2024, respectively, which are indicated exclusively for preventing lower respiratory diseases caused by RSV infection in adults over 60 years (15–18).

Although these preventive interventions demonstrate clinical efficacy, their cost-effectiveness has not been evaluated systematically across diverse healthcare systems. Given the emergence of new preventive interventions, it is crucial not only to assess their economic feasibility but also to understand the models describing their impact. This study aims to conduct a comprehensive systematic review of the economic impact of current and emerging RSV prevention strategies, integrating economic evaluation with their modeling methodologies. The findings will provide evidence to inform decision-makers and health technology assessors, offering insights into both the practical and economic value of these interventions.

## 2 Methods

The protocol for this study has been registered with PROSPERO under the registration number CRD42024524720. This study adheres to the Preferred Reporting Items for Systematic Reviews and Meta-Analyses (PRISMA) checklist for systematic (19).

### 2.1 Search strategy

For this systematic review and meta-analysis, a comprehensive search was conducted across PubMed, Embase, the Cochrane Library, Web of Science, and Tufts Registry, covering all studies published up to 12 March 2025. We utilized a combination of six terms and their synonyms, using both Medical Subject Headings (MeSHs) and free-text terms: respiratory syncytial virus, respiratory tract infection, vaccine, vaccination, monoclonal antibody, cost-effectiveness analysis, cost–benefit analysis, cost-utility analysis. The PubMed search strategy outlined in Supplementary Table S1 can be adapted for other databases.

### 2.2 Study selection

The inclusion and exclusion criteria are based on the PICOS principles. The inclusion criteria are as follows: (1) Population: Infants, children, older adults, immunocompromised individuals, or other at-risk populations for RSV infection; (2) Intervention: Any preventive strategy for RSV, such as maternal vaccines, pediatric vaccines, monoclonal antibodies, or passive immunization; (3) Comparator: No intervention, placebo, or alternative RSV prevention strategies; (4) Outcomes: Economic outcomes, including cost-effectiveness ratios (e.g., ICER), cost per quality-adjusted life year (QALY) or disability-adjusted life year (DALY), and cost–benefit analysis; (5) Study Type: Full economic evaluations, including cost-effectiveness analysis, cost–benefit analysis, or cost-utility analysis.

The exclusion criteria are defined as follows: (1) Studies that do not perform an economic evaluation; (2) Studies focusing on clinical outcomes without any economic analysis; (3) Studies that do not focus on RSV prevention strategies; (4) Non-peer-reviewed sources, abstracts, commentaries, and editorials.

### 2.3 Data extraction

The standardized data extraction tool was developed based on the Consolidated Health Economic Evaluation Reporting Standard checklist (CHEERS) (20), with two formats modified to capture both cost-effectiveness analysis (CEA) and cost–benefit analysis (CBA).

Two reviewers (YZ and WL) independently extracted the data, and any inconsistencies were then settled through discussion. Any disagreements were discussed and resolved with a third researcher (BZ). The extracted data comprised title, authors, years of publication, setting for the economic evaluation, type of intervention, comparator, type of economic evaluation, perspective, type of model, discount rate, currency and year of value, the type of sensitivity analysis and the most sensitive parameter, the incremental cost and incremental outcomes or Benefit cost ratio/Net present value and the author’s conclusion. When detailed information on outcomes was incomplete, we contacted the study authors directly. For studies with incomplete or unclear reporting of ICER components, such as unspecified cost categories or utility weights, we recorded the available information and explicitly noted the missing elements. These studies were included in the qualitative synthesis but were not used for direct cross-study comparisons of ICER values.

The included studies differed in characteristics such as time of the conduct, type of economic evaluation (EE) used, model type, time horizon, perspective, and expression of economic outcomes. Therefore, the outputs were not synthesized using meta-analysis. Nevertheless, decision-makers will find a descriptive synthesis informative in identifying the scope and quality of relevant studies, while showing the impact of the main parameters on the overall result (21).

Descriptive statistics were used to summarize publication, study, and population attributes, e.g., study design, publication details, and outcome measures. All screening and summary statistics were performed using Microsoft Excel.

### 2.4 Risk of bias assessment and reporting quality assessment

The risk of bias in each included study was assessed using the Economic Evaluations Bias (ECOBIAS) checklist (22). The ECOBIAS checklist includes a total of 22 biases organized under two main parts (Part A and Part B). Part A consists of 11 items for assessing an overall bias in economic evaluation. Part B, which also consists of 11 items, helps to assess model-specific aspects of bias in economic evaluation. Each item was graded as yes, partly, or no.

The reporting quality of the included studies was assessed using the revised version of the Consolidated Health Economic Evaluation Reporting Standards (CHEERS) 2022 statement (21). The new CHEERS checklist contains 28 items which are subdivided into seven main categories: (i) Title, (ii) Abstract, (iii) Introduction, (iv) Methods, (v) Results, (vi) Discussion, and (vii) Other Relevant Information, with each item rated as yes, partly, or no (21). As the CHEERS checklist is used to assess the quality of reporting of EE studies rather than the quality of its conduct, we performed a qualitative assessment of reporting completeness for each item of included studies (21).

This study assigned a value of 1 to “Yes,” 0.5 to “Partly,” and 0 to “No,” and then calculated the total scores for each study across the two scales to quantify their respective risk of bias and quality.

## 3 Results

### 3.1 Search results

**Figure 1:**
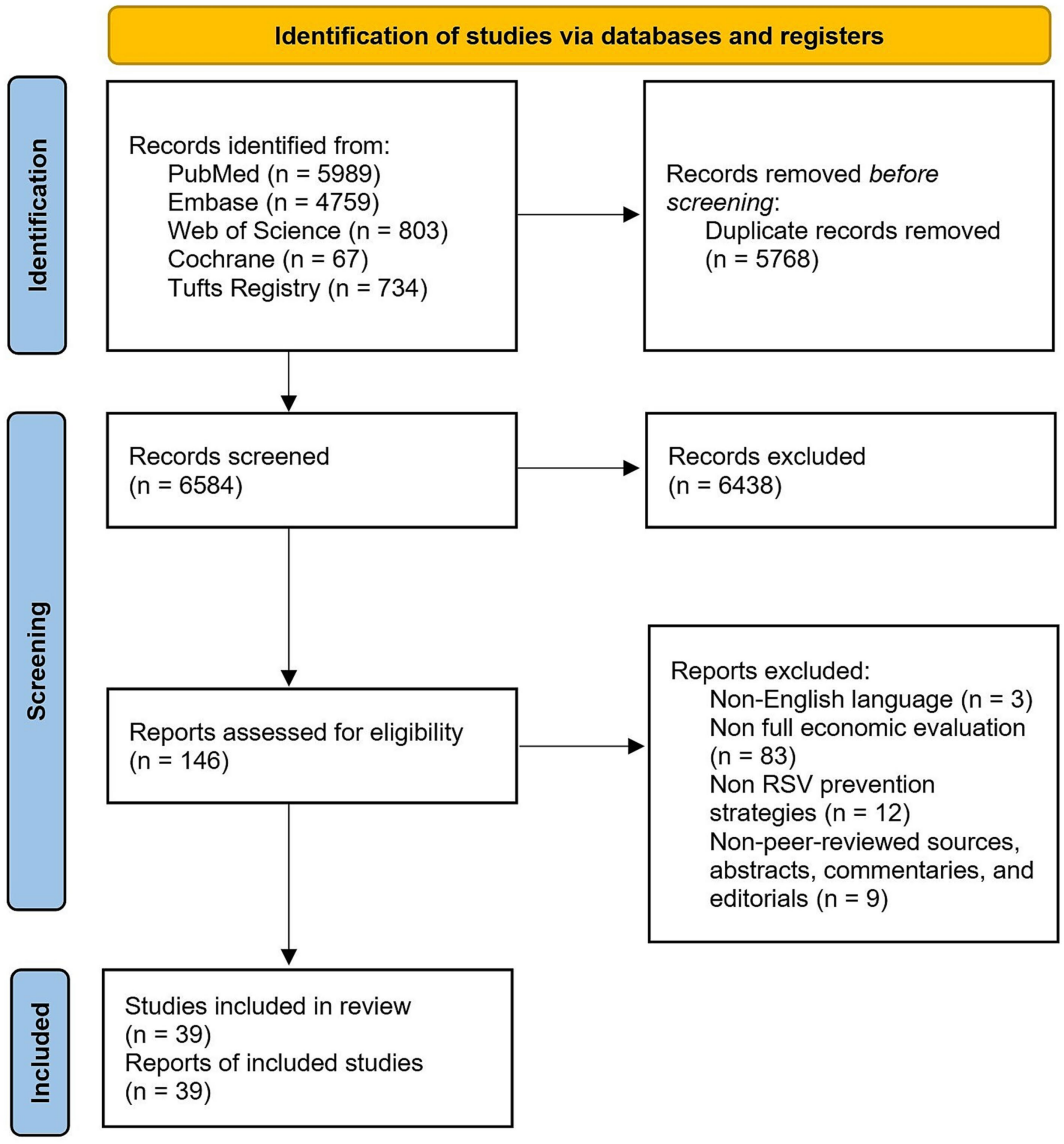
PRISMA flow diagram.

The search in four databases yielded a total of 12,352 articles, out of which 5,768 duplicates were removed. The remaining 6,584 articles were screened by title and abstract, of which 6,438 articles were excluded as they did not meet the inclusion criteria, leaving 146 studies for full-text evaluation. Out of the 146 studies, 39 studies were included in our analysis after 107 studies were excluded for the following reasons: non-English language (n = 3), non-full economic evaluation (n = 83), non-RSV prevention strategies (n = 12), non-peer-reviewed sources, abstracts, commentaries, and editorials (n = 9). The detailed search algorithms are provided in Figure 1. Notably, two modeling comparison studies were retained in the analysis (23, 24), as they generated utilizable outcomes that met the inclusion criteria.

### 3.2 Study characteristics

An overview of the study characteristics is presented in Supplementary Table S2. The 39 studies reported analyses from different countries and continents (Table 1). Most studies were conducted in high-income countries (HICs) (25–34), and only 8 studies were conducted in lower middle-income countries (LMICs) (35–41). There was only one cost–benefit analysis (CBAs) (25), while others were all cost-effectiveness analyses (CEAs). In these CEAs, there were 31 studies using QALY to measure health benefits, which can be classified as cost-utility analyses (CUAs) (26, 28–31, 33, 36, 42–45). Studies adopted either healthcare system perspective or societal perspective, with 10 studies reporting results from both perspectives concurrently. The investigated populations encompassed infants/children, older adults, and pregnant women (along with their neonates). The interventions covered four currently approved RSV prevention modalities (AREXVY®, ABRYSVO®, nirsevimab, and palivizumab). All of the studies used current practices as comparators. Where multiple strategies were assessed, incremental analysis was undertaken in such a way that each strategy was compared with the others in terms of costs, after identifying dominated options.

**Table 1:** Summary of characteristics of research articles.

Category	Subcategory	Studies
Country/regions	Canada	7
	USA	5
	UK	4
	Japan	2
	Mali	2
	Argentina	2
	China	2
	Other countries	14
Income level	HIC	31
	LMIC	8
Study type	CBA	1
	CEA	7
	CUA	31
Study perspective	Healthcare system	22
	Societal	20
	Not stated	7
Interventions	Vaccine-related	36
	mAb-related	18

HIC, high-income countries; LMIC, lower middle-income countries; CBA, cost–benefit analysis; CEA, cost-effectiveness analysis; CUA, cost-utility analysis; QALY, quality-adjusted life year; DALY, disability-adjusted life year.

### 3.3 Model design

The model designs and input parameters are presented in Table 2, with more detailed extraction tables available in Supplementary Table S3. All but four studies reported comprehensive descriptions of model types and structures, including decision tree model, Markov model, existing models (McMarcel or UNIVAC), discrete-event simulation, dynamic transmission model, individual-based model, and other models.

**Table 2:** Model design and inputs of research articles.

Category	Subcategory	Studies
Model type	Decision Tree	9
	Markov Model	12
	Discrete-event Simulation	4
	Dynamic Transmission Model	2
	Individual-based Model	1
	Other Models (including UNIVAC and McMarcel)	7
	Not stated	4
Time horizon	10 Years or More	9
	1 Year to 10 Years	9
	1 Year or Less	9
	Not stated	12
Discount Rate	1.5–5%	23
Health Outcomes	QALY	31
	DALY	8
	Per clinical event avoided	2
WTP Threshold Reported	Yes	28
	No	11

QALY, quality-adjusted life year; DALY, disability-adjusted life year, WTP, willingness-to-pay.

Decision-analytic tree models was implemented for analyses with limited time horizons (1–3 years), incorporating two primary nodal states: medically-attended symptomatic RSV versus non-symptomatic cases, with subsequent stratification by healthcare utilization levels, ranging from primary care consultations to intensive care unit hospitalizations (42, 46, 47). Wang et al. (46) extended this framework to include vaccination-related adverse outcomes as a distinct state while differentiating RSV clinical manifestations as RSV LRTD and RSV ARI.

Markov models were predominantly employed in long-term studies [extending up to lifetime horizons (26, 48–50)] and were frequently combined with decision-tree architectures. While sharing similar fundamental health states with decision-tree models, several studies implemented unique state elaborations leveraging Markovian properties. Mizukami et al. (51) introduced three distinct reinfection states (reinfection with RSV, reinfection with RSV-LRTD, and reinfection with RSV-URTD). La EM et al. (52) incorporated a “Post-RSV” health state representing recovery from both RSV-LRTD and RSV-URTD, while accounting for recurrent infections within their model framework. Pouwels et al. (41) added a “Susceptible” state preceding RSV infection to explicitly differentiate disease susceptibility prevalence from general population incidence rates.

The four discrete-event simulation model studies featured relatively short time horizons (1–2 years), yet demonstrated remarkable structural consistency. In addition to the health states shared with the aforementioned model types, all DES studies incorporated a “Recovered” state, analogous to La et al.’s (52) “Post-RSV” state. Notably, Moghadas et al. (44) uniquely included “Mechanical ventilation” as a distinct model state among all reviewed studies, justified by the clinical finding that 16.6% of ICU-admitted patients required ventilatory support.

The two dynamic transmission models, developed by the same research team, employed identical study timeframes and model architectures. These models stratified the population into six different epidemiological states (M: protected due to maternal antibodies, S: susceptible, E: exposed but not infectious, I: infectious and symptomatic, A: infectious and asymptomatic, R: recovered and protected). This represents an expansion of the classical SEIR (Susceptible-Exposed-Infectious-Recovered) framework through the addition of two novel compartments: (1) maternal antibody-mediated protection (M) and (2) asymptomatic infectious status (A).

The open-access UNIVAC (53) and McMarcel (39) models provide complementary RSV evaluation approaches. UNIVAC, an Excel-based and universal vaccine platform, tracks cases, visits, hospitalizations, and deaths through adaptable modules applicable to multiple diseases, while McMarcel specifically analyzes maternal/neonatal RSV immunization in 72 Gavi countries using a streamlined three-state framework (symptomatic infection, no symptoms, death). UNIVAC enables broad vaccine comparisons through customizable parameters, whereas McMarcel delivers pre-parameterized policy analysis for LMIC settings, with both tools undergoing extensive validation.

### 3.4 Model inputs

All but four studies (30, 37, 47, 54) reported discount rates, which ranged from 1.5 to 5%, with the lowest rates observed in Canadian studies and the highest rates in Mexican and Australian research. Health outcome measures were universally reported, with all CUAs (26, 28–31, 33, 36, 42–45) employing QALYs, while other CEAs utilizing DALYs (32, 36–39, 55). Additionally, two studies reported cost per hospitalized RSV case avoided and cost per life year gained, respectively. Detailed costs were documented in all studies, uniformly including direct medical costs. Supplementary Table S3 displayed complete cost structures. Willingness-to-pay (WTP) thresholds were reported in all but 11 studies (24, 25, 27, 29, 34, 39, 49, 51, 56), with most thresholds deriving from local guidelines.

The cost inputs were intrinsically linked to the adopted analytical perspective, with comprehensive cost breakdowns provided in Supplementary Table S3. Studies adopting the healthcare system perspective uniformly included direct medical costs encompassing two primary categories: (1) vaccine or monoclonal antibody (mAb)-related expenses, incorporating the procurement costs of biological products, administration fees, and adverse event management expenditures (46, 51), and (2) healthcare utilization costs across various treatment settings, including hospitalization expenses (both general ward and ICU admissions), outpatient visit charges, and primary care consultation fees. Notably, four studies (35, 48, 50, 56) that ostensibly adopted the healthcare system perspective paradoxically incorporated indirect costs such as productivity losses and transportation expenses into their models—components typically excluded under this perspective. In contrast, studies employing the societal perspective systematically included both direct costs (with compositions similar to those described above) and indirect costs, the latter primarily consisting of out-of-pocket expenditures and parental productivity loss. Shoukat A et al. (57) extended this framework by additionally accounting for the monetary valuation of life years loss due to RSV-related infant mortality, thereby incorporating an extra dimension of economic burden.

### 3.5 Base-case results

Base case results are summarized in Table 3. A more detailed list of ICERs is compiled under Supplementary Table S4.

**Table 3:** Base case results of the economic evaluation of RSV preventive strategies.

Study	Benefit–cost ratio	ICER	Base case results
Thomas (25)	1.3906		Passive immunisation programme was a cost-saving choice.
Cromer et al. (42)			The MCEP for combination of a newborn and infant programme was £246 (95% UI 219–275).
Wang et al. (46)		ABRYSVO®: 137,907 USD/QALY AREXVY®: 219,299 USD/QALY	ABRYSVO® group was accepted as the cost-effective option at the 25% US vaccine price level.
Álvarez Aldean et al. (64)		Dominant	Maternal vaccination resulted in a dominant strategy compared to no intervention in the Spanish NHS setting.
Ishiwada et al. (48)		Payer perspective: 4,998,847¥/QALY Societal perspective: 4,638,509¥/QALY	A combination prophylaxis was cost-effective under the ICER threshold of ¥5 million per QALY.
Shoukat et al. (57)		Birth cohorts: 4200 CAD/QALY Pregnant women: 41,321 CAD/QALY	Nirsevimab would be cost-effective from a societal perspective for a PPD of up to $290.
Mizukami et al. (51)		4,180,084 JPY/QALY	Vaccination was cost-effective compared to no intervention.
Averin et al. (65)		36,064 €/QALY	RSVpreF has the potential to greatly reduce the public health and economic burden of RSV among older adults in Germany.
Gourzoulidis et al. (26)		19,723 EUR/QALY	RSVpreF was cost-effective in Greek adults over 60 years of age.
Rey-Ares et al. (35)			The price of RSVpreF was estimated to be $74.46 per dose.
Laufer et al. (36)		(Societal perspective) Short-acting mAb: 4164 USD /DALY Long-acting mAb: 1614 USD/DALY Maternal vaccine: 8038 USD/DALY	Long-acting monoclonal antibody is likely to be cost-effective from both government and donor perspectives at $3 per dose.
Hutton et al. (27)		396,280$/QALY	RSVpreF has the potential to be cost-effective in specific circumstances, particularly when administered at the ideal gestational and seasonal time.
Bugden et al. (47)		(cost per averted hospitalisation) NIRS HR: Cost saving NIRS HR + MR: $8,139 ABR SEASONAL: $23,896 ABR SEASONAL + NIRS: $23,790 ABR ALL: $36,396 ABR ALL + NIRS: $36,378 NIRS < 6: $49,683 NIRS ALL: $53,113	Targeted administration of Nirsevimab to high-risk infants demonstrates superior cost-effectiveness compared to palivizumab in southern Canada.
Meijboom et al. (43)		34,143€/QALY	Vaccination of infants against RSV might be cost-effective.
Moghadas et al. (44)		①Arexy: 93981$/QALY ②Abrysvo: 94651$/QALY ③Arexy and Abrysvo:94234$/QALY	Vaccination programs could be cost-effective for a PPD up to $127 with Arexvy and $118 with Abrysvo over the first RSV season under the WTP of $95,000 per QALY gained.
Li et al. (59)			At NOK 500 per dose, mAb “Nov-Feb,” “Oct-Feb” or “Oct-Mar” is the cost-effective intervention.
Li et al. (56)		Payers perspective MV: UA: 402349€/QALY NV: 463979€/QALY SPS: 366437€/QALY SPD: 1973816€/QALY LSHTM: 178322€/QALY mAb: UA: 71522€/QALY NV: 69419€/QALY SPS: 61626€/QALY SPD: 101282€/QALY LSHTM: 54272€/QALY societal perspective MV: UA: 332952€/QALY NV: 375702€/QALY SPS: 297665€/QALY SPD: 1901299€/QALY LSHTM: 162266€/QALY mAb: UA: 11658€/QALY NV: Dominated SPS: 1635€/QALY SPD: 34327€/QALY LSHTM: 35205€/QALY	The LSHTM model had the highest QALY losses due to RSV episodes because it attributed QALY losses to non-MA symptomatic RSV infections. The SPD model reported the lowest QALY losses due to RSV episodes because it estimated lower incidences for both MA and non-MA symptomatic infections.
Rudd et al. (23)			Vaccination for people with CMCs over the age of 70 years was the optimal cost-effectiveness strategy when using a threshold of $50,000/QALY.
Gebretekle et al. (24)		Seasonal nirsevimab for infants at moderate or high risk, no catch-up: Dominated Seasonal nirsevimab for infants at moderate or high risk, with catch-up: 27891$/QALY Year-round nirsevimab for infants at moderate or high risk: Dominated Year-round RSVpreF plus nirsevimab for infants at high-risk: 204621$/QALY Year-round RSVpreF for all pregnant women and pregnant people: Dominated Seasonal nirsevimab for all infants, no catch-up: Dominated Seasonal nirsevimab for all infants, with catch-up: 512265$/QALY Year-round nirsevimab for all infants: Dominated	Seasonal nirsevimab for infants at moderate- and high-risk with catch-up was the most cost-effective strategy with an ICER of $27, 891 per QALY when compared to palivizumab.
Liu et al. (60)			Year-round infant mAb plus paediatric immunisation is the most cost-effective among all the year-round strategies.
La et al. (52)		18,430$/QALY	Adjuvanted RSVPreF3 vaccination is a cost-effective option for the prevention of RSV in US adults aged ≥ 60 years.
Hutton et al. (49)		Vaccine vs. no vaccine ($/QALY) GSK: ① 60–65: 385829 ② 65–70: 253967 ③ 70–75: 233472 ④75–80: 92438 ⑤ ≥80: 110830 Pfizer: ① 60–65: 331486 ② 65–70: 225521 ③ 70–75: 207453 ④ 75–80: 84652 ⑤ ≥80: 100726	Vaccination in adults aged ≥60 years may be cost-effective, particularly in those at more advanced age.
Huerta et al. (50)		Maternal vaccine: $247102million/QALY	Year-round RSVpreF maternal vaccination would likely represent a cost-effective program.
Nourbakhsh et al. (54)		Palivizumab: 1011139$/QALY LAMA: 883539$/QALY Maternal vaccine: 227286$/QALY Maternal vaccine + LAMA for preterm and chronically ill infants: 204621$/QALY	Palivizumab offered to full-term infants aged 0–2 months and high-risk for complicated RSV disease is not cost-effective, compared to preterm/chronically ill infants under 1 year of age.
Nazareno et al. (28)		maternal RSV vaccination program: 11403.10$AU/QALY	Maternal vaccination can be cost-effective up to $AU 120 per dose compared to no vaccination at WTP of $AU 50,000 per QALY gained.
Koltai et al. (37)			Interventions against RSV disease may be more cost-effective than previously estimated.
Hodgson et al. (29)			Long-acting monoclonal antibody programme was cost-effective.
Guiñazú et al. (38)			Long-acting monoclonal antibody and maternal RSV vaccine are both cost-effective compared to no intervention.
Zeevat et al. (30)			Justifiable vaccine prices of €16.38 and €50.03 were found based on the application of the lower and higher WTP thresholds, respectively.
Li et al. (39)			The maternal strategy is the most cost-effective strategy in LMICs (1000–8,000 USD per DALY).
Shoukat et al. (31)		$49,653/QALY	Arexvy would be cost-effective from a societal perspective when vaccinated to 90% of residents in LTCHs for a PPD up to $163 at a WTP of $50, 000 per QALY gained.
Baral et al. (32)		$1,342/DALY	Maternal vaccine and mAbs were cost-effective in 60 and 118 countries at a 50% gross domestic product per capita threshold, respectively.
Do et al. (40)		$3,442/DALY	Both RSVpreF and Nirsevimab have the potential to be cost-effective.
Laufer et al. (55)		$597/DALY	Extended half-life RSV mAbs would be impactful and efficient components of prevention strategies in LMICs such as Mali.
Pouwels et al. (41)		51969TL/QALY	All strategies remained slightly below the threshold of 3 times the GDP per capita.
Hodgson et al. (33)			LAMA could be cost-effective for up to £84 CCPA and MV could be cost-effective for up to £80 CCPA.
Tuite et al. (45)			Vaccinating adults aged 70 years and older with 1 or more chronic medical condition was the optimal strategy for a cost effectiveness threshold of $50,000 per QALY.
Meijboom et al. (58)		€133,068/QALY	Vaccination of the complete 60 + older adult cohort would not be cost-effective.
Gessner et al. (34)		$5,342/QALY	RSV vaccine would be cost-effective for the older adults, with cost-effectiveness ratios similar to those for influenza vaccine.

RSV, respiratory syncytial virus; ICER, incremental cost effectiveness ratio; QALY, quality-adjusted life years; DALY, disability adjusted life years; WTP, willingness-to-pay; LAMA, long-acting monoclonal antibodies; mAb, monoclonal antibodies; MA, medically attended; PPD, price-per-dose; CCPA, could be cost-effective for purchasing and administration; NHS, national healthcare system.

#### 3.5.1 Studies reporting benefit–cost ratios

Only one study reported benefit–cost ratio (BCR) (25). A BCR value greater than 1.0 shows that the intervention is expected to deliver a positive net present value.

The economic evaluation demonstrated that compared to emergency inpatient admissions for RSV, implementing an infant immunization program with palivizumab for cases of extreme immaturity (EI) would generate substantial cost savings. The analysis estimated £50,780,109.02 in potentially avoidable RSV-related costs, compared to £36,516,391.94 in total immunization program costs, which yielded a favorable BCR of 1.39 and net savings of £14,263,717.08. These findings robustly indicate that palivizumab immunization for this high-risk infant population represents a cost-saving preventive strategy against RSV disease burden.

#### 3.5.2 Studies reporting cost per QALY or DALY

Twenty-four studies reported cost per QALY or DALY, with their base-case analyses yielding varied conclusions (24, 26–28, 31, 41, 43, 44, 46, 52, 56, 58). The economic evaluation results were presented in multiple formats: (1) as raw values, including the incremental cost-effectiveness ratio (ICER), expressed as the incremental cost per QALY or DALY gained; (2) through comparison of ICER results against local WTP thresholds for each QALY to determine cost-effectiveness; or (3) as dominated alternatives, characterized by both higher QALYs and lower costs compared to the comparator intervention.

For older adults, ABRYSVO® and AREXVY® demonstrated varying ICERs across the US and other high-income countries (27, 28, 44, 49, 51, 52), with all studies confirming the economic superiority of vaccination versus no vaccination or standard interventions. Tuite AR et al. (45) conducted a modeling study involving a multi-age cohort of 100,000 individuals aged ≥50 years, with stratification by both age and risk profile. Their analysis identified vaccination of adults aged ≥70 years with one or more chronic medical conditions as the optimal intervention strategy when applying a cost-effectiveness threshold of $50,000 per QALY, aligning with Rudd et al.’s results (38).

.In neonatal prevention, primarily comparative analyses between two monoclonal antibody products and maternal vaccination, demonstrated superior cost-effectiveness of LAMA. Li et al. (39) found that “mAb” strategy is more effective due to its assumed longer duration of protection versus maternal vaccination, but it was also assumed to be more expensive. Laufer et al. (36) found that LAMA achieved the lowest cost per DALY, followed by short-acting monoclonal antibody, with maternal vaccine being the least cost-effective option. This conclusion was further supported by Li et al. (56), who used cost per QALY as the metric.

However, Nourbakhsh et al. (54) stressed that both the maternal vaccine and mAb strategies need to be competitively priced to be judged as relatively cost-effective. Otherwise, these interventions would only be cost-effective when targeting high-risk populations (e.g., preterm infants or infants <1 year with chronic conditions), while Hutton et al. (27) also demonstrated that maternal vaccination can be more cost-effective if administered to pregnant women immediately before or during the RSV season. These findings collectively indicate that the economic attractiveness of RSV prevention strategies is significantly influenced by multiple factors, including vaccine price, target population characteristics, and timing of administration relative to the RSV season.

#### 3.5.3 Studies reporting cost per event averted

Only two studies (26, 47) specifically presented cost per clinical event averted, both using RSV-related hospitalization as the defined clinical endpoint. For example, Bugden et al. (47) calculated the cost of averting one hospitalisation for each new strategy compared to no intervention, finding that interventions were always cost-saving in Nunavut and Nunavik. But only under strictly conditional administration would nirsevimab and RSVpreF demonstrate cost-saving potential in the Northwest Territories and southern Canada, while a broader prophylactics implementation requires an expenditure of $6,247 to $53,113 per hospitalization prevented.

### 3.6 Sensitive analysis results

The type of sensitivity analyses conducted and the results of the sensitivity analysis are reported in Supplementary Table S5. Deterministic sensitivity analyses (DSA) uniformly identified the top three most influential parameters, while probabilistic sensitivity analyses (PSA) reported the probability of interventions being cost-effective at specified thresholds, with scenario analysis configurations detailed in Supplementary Table S5. All but four studies (23, 29, 44, 54) comprehensively reported sensitivity analysis results, 13 studies presenting only DSA results (24, 25, 32, 33, 36, 39, 42, 48, 49, 56, 58–60), 3 studies reporting exclusively PSA findings (34, 37, 57), and the remaining 19 studies incorporating both analytical approaches.

Substantial heterogeneity emerged in the identified sensitive parameters across studies. Vaccine efficacy was reported among the three most sensitive parameters in over half of the studies. Other frequently influential parameters included intervention price, protection duration, hospitalization incidence etc.

### 3.7 Risk of bias of the studies

The methodological quality assessment using ECOBIAS checklists yielded an average score of 16.6 across all studies (range: 10.5 (29) to 20 (45)), indicating generally low risk of bias in the included economic evaluations. However, three prevalent bias domains were identified in nearly all studies: (1) reporting and dissemination bias, (2) bias related to treatment effects, and (3) bias concerning internal consistency. Table 4 shows detailed 22 items results.

**Table 4:** Results of 22-items from the ECOBIAS checklists.

Study	1	2	3	4	5	6	7	8	9	10	11	12	13	14	15	16	17	18	19	20	21	22	Total
Thomas (25)	P	Y	Y	Y	Y	N	P	Y	Y	Y	N	N	Y	N	Y	Y	N	N	N	Y	Y	N	13
Cromer et al. (42)	N	Y	Y	Y	Y	N	P	Y	Y	Y	N	N	Y	N	Y	Y	N	N	N	Y	Y	N	12.5
Wang et al. (46)	N	Y	Y	Y	Y	Y	P	Y	Y	Y	N	Y	Y	Y	Y	Y	Y	Y	Y	Y	Y	N	18.5
Álvarez Aldean et al. (64)	N	Y	Y	Y	Y	Y	P	Y	Y	Y	N	Y	Y	Y	Y	Y	P	N	Y	Y	Y	N	17
Ishiwada et al. (48)	N	Y	Y	Y	Y	Y	P	Y	Y	Y	N	Y	Y	Y	Y	Y	Y	N	Y	Y	Y	N	17.5
Shoukat et al. (57)	Y	Y	Y	Y	Y	Y	P	Y	Y	Y	N	Y	Y	Y	Y	Y	Y	N	Y	Y	Y	N	18.5
Mizukami et al. (51)	N	Y	Y	Y	Y	Y	P	Y	Y	Y	N	Y	Y	Y	Y	Y	Y	Y	Y	Y	Y	N	18.5
Averin et al. (65)	Y	Y	Y	Y	Y	Y	P	Y	Y	Y	N	P	Y	P	Y	Y	P	Y	Y	Y	Y	N	18
Gourzoulidis et al. (26)	Y	Y	Y	Y	Y	Y	P	Y	Y	Y	N	P	Y	P	Y	Y	P	N	Y	Y	Y	N	17
Rey-Ares et al. (35)	N	Y	Y	Y	Y	Y	P	Y	Y	Y	N	Y	Y	Y	Y	Y	Y	N	Y	Y	Y	N	17.5
Laufer et al. (36)	Y	Y	Y	Y	Y	P	P	Y	Y	Y	N	Y	Y	Y	Y	Y	Y	N	N	Y	Y	N	17
Hutton et al. (27)	Y	Y	Y	Y	Y	P	P	Y	Y	Y	N	Y	Y	Y	Y	Y	Y	Y	P	Y	Y	N	18.5
Bugden et al. (47)	Y	Y	Y	Y	Y	Y	P	Y	Y	Y	N	Y	Y	Y	Y	Y	Y	N	Y	Y	Y	N	18.5
Meijboom et al. (43)	Y	Y	Y	Y	Y	Y	P	Y	Y	N	N	N	Y	N	Y	Y	N	N	Y	Y	Y	N	14.5
Moghadas et al. (44)	Y	Y	Y	Y	Y	Y	P	Y	N	Y	N	Y	Y	Y	Y	Y	P	N	N	Y	N	N	15
Li et al. (59)	N	Y	Y	Y	Y	Y	P	Y	Y	Y	N	Y	Y	Y	Y	Y	P	N	Y	Y	Y	N	17
Li et al. (56)	Y	Y	Y	Y	Y	Y	P	Y	Y	Y	N	N	Y	N	Y	N	N	N	Y	Y	Y	Y	15.5
Rudd et al. (23)	N	Y	Y	Y	N	Y	P	Y	Y	N	N	N	Y	N	Y	Y	N	Y	Y	Y	Y	Y	14.5
Gebretekle et al. (24)	Y	Y	Y	Y	Y	Y	P	Y	Y	Y	N	Y	Y	Y	Y	Y	Y	Y	Y	Y	Y	N	19.5
Liu et al. (60)	Y	Y	Y	Y	Y	Y	P	Y	Y	Y	N	Y	Y	Y	Y	Y	Y	N	Y	Y	Y	N	18.5
La et al. (52)	Y	Y	Y	Y	Y	Y	P	Y	Y	Y	N	Y	Y	Y	Y	N	Y	Y	Y	Y	Y	N	18.5
Hutton et al. (49)	Y	Y	Y	Y	Y	Y	P	Y	Y	Y	N	N	Y	N	Y	Y	N	N	N	Y	Y	N	14.5
Huerta et al. (50)	N	Y	Y	Y	Y	Y	P	Y	Y	Y	N	Y	Y	Y	Y	N	P	Y	Y	Y	Y	N	17
Nourbakhsh et al. (54)	N	Y	Y	Y	Y	Y	P	N	N	Y	N	N	Y	N	Y	Y	N	N	Y	Y	N	N	11.5
Nazareno et al. (28)	N	Y	Y	Y	Y	Y	P	Y	Y	Y	N	Y	Y	Y	Y	Y	Y	N	N	Y	Y	N	16.5
Koltai et al. (37)	N	Y	Y	Y	Y	Y	P	N	Y	Y	N	N	Y	N	Y	Y	N	N	Y	Y	Y	N	13.5
Hodgson et al. (29)	N	Y	Y	Y	Y	Y	P	Y	N	Y	N	N	Y	N	Y	N	N	N	N	Y	N	N	10.5
Guiñazú et al. (38)	Y	Y	Y	Y	Y	Y	Y	Y	Y	Y	N	P	Y	Y	Y	Y	Y	N	Y	Y	Y	N	18.5
Zeevat et al. (30)	Y	N	Y	Y	Y	Y	Y	N	Y	Y	N	Y	N	Y	Y	P	N	N	Y	Y	Y	N	14.5
Li et al. (39)	N	N	Y	Y	Y	Y	P	Y	Y	Y	N	Y	N	Y	Y	Y	Y	Y	Y	Y	Y	N	16.5
Shoukat et al. (31)	Y	Y	Y	Y	Y	Y	Y	Y	Y	Y	N	Y	Y	Y	Y	Y	Y	N	Y	Y	Y	N	19
Baral et al. (32)	N	Y	Y	Y	Y	Y	Y	Y	Y	Y	N	P	Y	Y	Y	Y	Y	N	Y	Y	Y	N	17.5
Do et al. (40)	Y	Y	Y	Y	Y	Y	Y	Y	Y	Y	N	Y	Y	Y	Y	Y	Y	N	Y	Y	Y	N	19
Laufer et al. (55)	Y	Y	Y	Y	Y	Y	P	Y	Y	Y	N	P	Y	Y	Y	Y	Y	N	Y	Y	Y	N	18
Pouwels et al. (41)	Y	N	Y	Y	Y	Y	Y	Y	Y	Y	N	Y	Y	Y	Y	Y	Y	N	Y	Y	Y	N	18
Hodgson et al. (33)	N	Y	Y	Y	Y	N	Y	Y	Y	Y	N	Y	Y	Y	Y	Y	Y	N	N	Y	Y	N	15
Tuite et al. (45)	Y	Y	Y	Y	Y	Y	Y	Y	Y	Y	N	Y	Y	Y	Y	Y	Y	N	Y	Y	Y	Y	20
Meijboom et al. (58)	N	N	Y	Y	Y	Y	Y	Y	Y	N	N	P	N	Y	Y	Y	Y	N	Y	Y	Y	N	14.5
Gessner et al. (34)	Y	N	Y	Y	Y	Y	P	Y	Y	Y	N	Y	N	Y	Y	Y	Y	N	Y	Y	Y	N	16.5

Y, yes; P, partly; N, no.

### 3.8 Quality of the studies

The methodological quality assessment using CHEERs checklists yielded an average score of 20.6 across all studies [range: 15.5 (42) to 23.5 (51)], suggesting generally high reporting quality. However, most studies failed to adequately present health economic analysis plan, characterizing distributional effects, approach to engagement with patients and others affected by the study, and effect of engagement with patients and others affected by the study. Table 5 shows detailed 28 items results.

**Table 5:** Results of 28-items from CHEERs checklists.

Study	1	2	3	4	5	6	7	8	9	10	11	12	13	14	15	16	17	18	19	20	21	22	23	24	25	26	27	28	Total
Thomas (25)	Y	N	Y	N	N	Y	Y	N	N	Y	P	Y	N	Y	Y	N	Y	N	N	Y	N	Y	Y	Y	N	Y	Y	Y	16.5
Cromer et al. (42)	Y	Y	Y	N	N	Y	Y	N	N	N	P	Y	N	Y	N	N	Y	N	N	Y	N	Y	Y	Y	N	Y	Y	Y	15.5
Wang et al. (46)	Y	N	Y	N	Y	Y	Y	Y	Y	Y	P	Y	Y	Y	Y	Y	Y	N	N	Y	N	Y	Y	Y	N	Y	Y	Y	21.5
Álvarez Aldean et al. (64)	Y	Y	Y	N	P	Y	Y	Y	Y	Y	P	Y	Y	Y	Y	Y	Y	Y	N	Y	N	Y	Y	Y	N	Y	Y	Y	23
Ishiwada et al. (48)	Y	Y	Y	N	N	Y	Y	Y	Y	Y	P	Y	Y	Y	Y	Y	Y	Y	N	Y	N	Y	Y	Y	N	Y	Y	Y	22.5
Shoukat et al. (57)	Y	Y	Y	N	N	Y	Y	Y	Y	Y	P	Y	Y	Y	Y	Y	Y	Y	N	N	N	N	Y	N	N	Y	Y	Y	19.5
Mizukami et al. (51)	Y	Y	Y	N	P	Y	Y	Y	Y	Y	Y	Y	Y	Y	Y	Y	Y	Y	N	Y	N	Y	Y	Y	N	Y	Y	Y	23.5
Averin et al. (65)	Y	Y	Y	N	P	Y	Y	Y	Y	Y	Y	Y	Y	Y	Y	P	Y	Y	N	Y	N	Y	Y	Y	N	Y	Y	Y	23
Gourzoulidis et al. (26)	Y	Y	Y	N	N	Y	P	Y	Y	Y	Y	Y	Y	Y	Y	P	Y	Y	N	Y	N	Y	Y	Y	N	Y	Y	Y	22
Rey-Ares et al. (35)	Y	N	Y	N	Y	Y	Y	Y	Y	Y	Y	Y	Y	Y	Y	Y	Y	Y	N	Y	N	Y	Y	Y	N	Y	Y	Y	23
Laufer et al. (36)	Y	Y	Y	N	N	Y	Y	Y	Y	Y	Y	Y	Y	Y	Y	Y	Y	Y	N	Y	N	Y	Y	Y	N	Y	Y	Y	23
Hutton et al. (27)	Y	Y	Y	N	N	Y	Y	Y	Y	Y	Y	Y	Y	Y	Y	Y	Y	N	N	Y	N	Y	Y	Y	N	Y	Y	Y	22
Bugden et al. (47)	Y	Y	Y	N	N	Y	Y	Y	Y	Y	P	Y	Y	Y	Y	Y	Y	Y	N	Y	N	Y	Y	Y	N	Y	Y	Y	22.5
Meijboom et al. (43)	Y	Y	Y	N	P	Y	Y	Y	N	Y	P	P	Y	Y	Y	P	Y	N	N	Y	N	Y	Y	Y	N	Y	N	N	18
Moghadas et al. (44)	Y	Y	Y	N	Y	Y	Y	Y	Y	Y	Y	Y	Y	Y	Y	Y	Y	N	N	Y	N	Y	Y	Y	N	Y	Y	Y	23
Li et al. (59)	Y	Y	Y	N	P	Y	Y	N	N	Y	Y	P	N	P	Y	Y	Y	N	N	Y	N	Y	Y	Y	N	Y	Y	Y	18.5
Li et al. (56)	Y	Y	Y	N	P	Y	Y	Y	Y	Y	P	Y	Y	P	Y	P	Y	Y	N	Y	N	Y	Y	Y	N	Y	Y	Y	22
Rudd et al. (23)	Y	Y	Y	N	Y	Y	Y	Y	Y	Y	P	Y	Y	P	Y	P	Y	N	N	N	N	Y	Y	Y	N	Y	N	N	18.5
Gebretekle et al. (24)	Y	Y	Y	N	P	Y	Y	Y	N	Y	Y	Y	Y	Y	Y	Y	Y	N	N	Y	N	Y	Y	Y	N	Y	Y	N	20.5
Liu et al. (60)	Y	Y	Y	N	P	Y	Y	Y	N	Y	Y	Y	N	Y	Y	Y	Y	Y	N	Y	N	Y	P	Y	N	Y	Y	N	20
La et al. (52)	Y	N	Y	N	Y	Y	Y	Y	Y	Y	P	P	Y	Y	Y	Y	Y	N	N	Y	N	Y	Y	Y	N	Y	Y	N	20
Hutton et al. (49)	Y	N	Y	N	Y	Y	Y	Y	Y	Y	P	P	Y	P	Y	P	Y	Y	N	Y	N	Y	Y	Y	N	Y	Y	N	20
Huerta et al. (50)	Y	N	Y	N	P	Y	Y	Y	Y	Y	P	Y	Y	Y	Y	Y	Y	N	N	Y	N	Y	Y	Y	N	Y	Y	Y	21
Nourbakhsh et al. (54)	Y	Y	Y	N	P	Y	Y	N	Y	N	P	Y	Y	Y	Y	P	Y	N	N	N	N	Y	Y	Y	N	Y	Y	N	17.5
Nazareno et al. (28)	Y	Y	Y	N	P	Y	Y	Y	Y	Y	Y	P	Y	Y	Y	Y	Y	N	N	Y	N	Y	Y	Y	N	Y	Y	N	21
Koltai et al. (37)	Y	Y	Y	N	Y	Y	Y	N	N	N	P	P	Y	Y	Y	P	Y	Y	N	Y	N	Y	Y	Y	N	Y	Y	N	18.5
Hodgson et al. (29)	Y	Y	Y	N	P	Y	Y	N	Y	Y	P	P	Y	Y	Y	P	Y	Y	N	P	N	Y	Y	Y	N	Y	Y	N	19.5
Guiñazú et al. (38)	P	Y	Y	N	Y	Y	P	Y	Y	Y	P	Y	N	Y	Y	P	Y	N	N	Y	N	Y	Y	Y	N	Y	Y	Y	20
Zeevat et al. (30)	N	Y	Y	N	P	Y	N	Y	Y	N	P	Y	N	Y	N	Y	Y	N	N	Y	N	Y	Y	Y	N	Y	Y	Y	17
Li et al. (39)	P	Y	Y	N	Y	Y	Y	Y	Y	Y	Y	Y	N	Y	Y	Y	Y	N	N	Y	N	Y	Y	Y	N	Y	Y	Y	21.5
Shoukat et al. (31)	P	Y	Y	N	P	Y	Y	Y	Y	Y	Y	Y	Y	Y	Y	Y	Y	Y	N	Y	N	Y	Y	Y	N	Y	Y	Y	23
Baral et al. (32)	P	Y	Y	N	Y	Y	Y	Y	Y	Y	P	Y	Y	Y	Y	P	Y	N	N	Y	N	Y	Y	Y	N	Y	Y	Y	21.5
Do et al. (40)	P	Y	Y	N	Y	Y	Y	Y	Y	Y	Y	Y	Y	Y	Y	Y	Y	N	N	Y	N	Y	Y	Y	N	Y	Y	Y	22.5
Laufer et al. (55)	Y	N	Y	N	P	Y	Y	Y	Y	Y	P	Y	Y	Y	Y	P	Y	N	N	Y	N	Y	Y	Y	N	Y	Y	Y	20.5
Pouwels et al. (41)	Y	Y	Y	N	P	Y	Y	Y	Y	Y	Y	Y	Y	Y	Y	Y	Y	N	N	Y	N	Y	Y	Y	N	Y	Y	Y	22.5
Hodgson et al. (33)	Y	Y	Y	N	P	Y	Y	Y	Y	Y	P	Y	Y	Y	N	Y	Y	N	N	Y	N	Y	Y	Y	N	N	Y	Y	20
Tuite et al. (45)	P	Y	Y	N	Y	Y	Y	Y	Y	Y	P	Y	Y	Y	Y	Y	Y	N	N	Y	N	Y	Y	Y	N	Y	Y	Y	22
Meijboom et al. (58)	P	Y	Y	N	P	Y	P	N	Y	Y	Y	Y	Y	Y	Y	P	Y	Y	N	Y	N	Y	Y	Y	N	Y	N	N	19
Gessner et al. (34)	P	N	Y	N	Y	N	P	Y	Y	Y	Y	Y	Y	Y	Y	Y	Y	N	N	Y	N	Y	Y	Y	N	Y	Y	N	19

Y, yes; P, partly; N, no.

## 4 Discussion

This systematic review evaluates and synthesizes economic assessments of current and emerging RSV prevention strategies, including vaccines and monoclonal antibody therapies, across different healthcare settings. Most studies found that preventive interventions using vaccines or monoclonal antibodies significantly reduce RSV-related hospitalization and mortality rates, though their cost-effectiveness varies across countries and populations. As prior studies have not specifically focused on the economic aspects of RSV vaccines, this study represents the first systematic review in this field.

Among currently available RSV vaccines, ABRYSVO® and AREXVY® demonstrate varying cost-effectiveness ratios (ICERs) in the United States and other high-income countries (27, 28, 44, 49, 51, 52), yet all studies consistently show that vaccination is more economically favorable than no vaccination or non-intervention measures. Notably, discrepancies emerged in two US-based societal perspective studies focusing on the economic evaluation of ABRYSVO® versus AREXVY® in older adults. Hutton et al. (49) demonstrated that ABRYSVO® had lower ICERs than AREXVY® across all five vaccination age groups examined (60–65, 65–70, 70–75, 75–80, and ≥80 years). In contrast, Moghadas et al. (44) found AREXVY® to be more cost-effective than ABRYSVO® (both individually and combined). This discrepancy could be attributed to Hutton DW et al.’s potential underestimation of ABRYSVO®'s efficacy (52.9% against hospitalization and 27.8% against outpatient care). Furthermore, Moghadas SM et al. utilized a discrete-event simulation model spanning only two RSV seasons, while Hutton DW et al. employed a lifetime horizon Markov model. Consequently, the ICER estimates were substantially higher in Hutton DW et al.’s findings.

In the monoclonal antibody strategies, nirsevimab and other LAMAs demonstrate favorable cost-effectiveness for high-risk infants due to their extended durability (24, 40, 47, 57). In certain low-income countries (e.g., Mali), short-acting antibodies such as palivizumab show significant socioeconomic benefits (36, 55). Gebretekle et al. (24) demonstrated that seasonal nirsevimab proves more cost-effective than palivizumab for moderate- to high-risk Canadian infants. The extended duration of protection, resulting in reduced annual administration frequency, may explain this economic advantage. This underscores the cost-saving advantage of long-acting monoclonal antibodies.

The existing evidence also supports the combination of vaccines and monoclonal antibodies for prevention. In Japan’s healthcare context, Ishiwada et al. (48) demonstrated that the combination of ABRYSVO® and palivizumab is a cost-effective choice compared to palivizumab alone from the payer perspective. Gebretekle et al. (24) provided evidence that Year-round ABRYSVO® for all pregnant women is more cost-effective than palivizumab. However, studies on the combination or comparison of RSV vaccines and monoclonal antibodies remain limited, which may become a future research direction for RSV prevention.

An important source of heterogeneity arises from the choice of economic modeling approach. Decision-analytic tree models, typically applied to short time horizons, tend to generate lower ICERs by simplifying long-term outcomes. In contrast, Markov models with lifetime horizons capture reinfections and long-term sequelae, often producing higher cost-effectiveness ratios. Discrete-event simulation models emphasize individual-level variability and can yield more conservative estimates in high-risk subgroups. Meanwhile, dynamic transmission models account for herd effects and indirect benefits, frequently enhancing the cost-effectiveness profile of interventions. These methodological differences highlight that model structure itself is a key determinant of ICER estimates.

Compared to other researches based on clinical trial data, three studies relied on model predictions with assumed vaccine efficacy (34, 43, 58). These studies were published before 2015 and were constrained by the availability of data. Due to the diversity of assumptions, they produced ICER results that varied significantly, and these results differed considerably from those based on clinical data. For example, Gessner et al. (34) produced an ICER of $5,342/QALY (43) in a study from a US societal perspective with no vaccination as the comparator. However, more recent empirical studies with the same setup report ICERs ranging from $18,430/QALY to $385,829/QALY (27, 44, 49, 52), indicating a significant disparity. This discrepancy may be attributed to the irrationality of certain assumptions or the rapid growth in healthcare prices, suggesting the need for caution in using predictions based on assumed efficacy and highlighting the necessity of updating empirical research.

Our study included research from both High-Income Countries (HICs) and Low- and Middle-Income Countries (LMICs), but the evidence from LMICs is significantly less than that from HICs. Our review indicates that nearly all studies from LMICs employed a cost-effectiveness threshold lower than the local per capita GDP (35–38, 40, 55), resulting in lower drug prices to achieve cost-effectiveness. The use of appropriate cost-effectiveness thresholds in low- and middle-income settings warrants further discussion. Similarly, as observed in previous studies on vaccines in low- and middle-income countries (61), we found considerable variation in the estimated costs of RSV hospitalization across diseases. In high-income settings (i.e., compared to LMICs), the cost per episode is generally higher, which may reflect greater healthcare expenses.

This systematic review has several limitations. First, inconsistent findings may exist across the included studies from various countries and regions due to differing interventions, study designs, and economic evaluation methods. For instance, the cost-effectiveness of nirsevimab may differ significantly between high-income and low-income countries (24, 40, 47, 57), indicating the need to consider and validate the results within specific economic and healthcare contexts. Second, most studies focus on short-term economic evaluations and overlook long-term health impacts, such as chronic respiratory diseases or asthma following RSV infection (2, 3, 61). These long-term effects could alter the cost-effectiveness conclusions. Finally, while most studies were from high-income countries, there were limited researches from low- and middle-income countries. More data and economic evaluations from these regions are needed, especially focusing on vaccine affordability, distribution, and social cost impacts (62, 63).

## 5 Conclusion

This systematic review examines the economic impact of RSV preventive interventions including RSV vaccines and mAb. This study highlights that most RSV vaccines and monoclonal antibody interventions are cost-effective, especially for high-risk groups. However, health technology assessors should pay particular attention to key factors that substantially influence cost-effectiveness, including the price, efficacy and duration, target population, and the administration timing of the intervention. Further research should prioritize the development of high-quality model-based economic evaluations for RSV prevention strategies, ensuring accessibility to decision-makers.

## Data Availability

The original contributions presented in the study are included in the article/Supplementary material, further inquiries can be directed to the corresponding author.
