# Supplementary material for: Economic evaluations of RSV preventive strategies: a systematic review of cost-effectiveness and modeling approaches
Source: Front Public Health. 2025 Oct 13;13:1672683. doi: 10.3389/fpubh.2025.1672683 (PMC12554770; doi:10.3389/fpubh.2025.1672683)
Supplement: Supplementary file 1 [file Table_1.docx]

**Table S1.** Search strategy (PubMed)

| # | Query (P+I+S) | Results |
| --- | --- | --- |
| 1 | "Respiratory Syncytial Viruses"[Mesh] | 11,650 |
| 2 | Respiratory Syncytial Virus OR Syncytial Virus, Respiratory OR Virus, Respiratory Syncytial OR RSV Respiratory Syncytial Virus OR Orthopneumovirus OR Orthopneumoviruses OR Chimpanzee Coryza Agent OR Chimpanzee Coryza Agents OR Coryza Agent, Chimpanzee | 21,542 |
| 3 | 1 OR 2 | 21,542 |
| 4 | "Respiratory Tract Infections"[Mesh] | 691,354 |
| 5 | Infection, Respiratory Tract OR Respiratory Tract Infection OR Infections, Respiratory OR Infections, Respiratory Tract OR Respiratory System Infections OR Infection, Respiratory System OR Respiratory System Infection OR Respiratory Infections OR Upper Respiratory Tract Infections OR Upper Respiratory Infections OR Upper Respiratory Tract Infection OR Infections, Upper Respiratory Tract OR Infections, Upper Respiratory OR Respiratory Infection, Upper | 781,779 |
| 6 | 4 OR 5 | 781,779 |
| 7 | 3 OR 6 | 785,156 |
| 8 | "Vaccines"[Mesh] OR "Vaccination"[Mesh] | 331,431 |
| 9 | Vaccine OR Vaccinations OR Immunization, Active OR Active Immunization OR Active Immunizations OR Immunizations, Active | 564,208 |
| 10 | 8 OR 9 | 564,208 |
| 11 | " Antibodies, Monoclonal"[Mesh] | 294,526 |
| 12 | Antibodies, Monoclonal OR Monoclonal Antibody OR nirsevimab [Supplementary Concept] OR palizumab | 402,293 |
| 13 | 11 OR 12 | 402,293 |
| 14 | 10 OR 13 | 947,858 |
| 15 | "Cost-Benefit Analysis"[Mesh] OR "Cost-Effectiveness Analysis"[Mesh] OR "Economics, Pharmaceutical"[Mesh] | 100,423 |
| 16 | cost benefit OR cost-benefit OR CBA OR benefit cost OR benefit-cost OR cost utility OR cost-utility OR CUA OR cost effectiveness OR cost-effectiveness OR CEA OR economic evaluation OR pharmacoeconomic OR pharmaco-economic OR economic model OR Markov model OR decision tree | 725,640 |
| 17 | 15 OR 16 | 725,640 |
| 18 | 7 AND 14 AND 17 | 5,989 |

**Table S2.** Studies included in the review and main study characteristics

| Study | Country | Perspective | Populations | Cohorts | Intervention | Comparator | Methods |
| --- | --- | --- | --- | --- | --- | --- | --- |
| Thomas G, 2015 | UK |  | Infants |  | Immunisation programme | No intervention | CBA |
| Cromer D, 2017 | UK | Health care provider | Infants, pregnant women, and neonates |  | (1) Vaccines;  (2) Prophylactic antibodies | No intervention | CEA, CUA |
| Wang Y, 2023 | China | Public healthcare Provider | ≥60y | 62y, 70y, 80y | Single RSV vaccination (AREXVY® or ABRYSVO®) | No intervention | CEA, CUA |
| Álvarez Aldean J, 2024 | Spain | National Healthcare System (NHS) | Pregnant women | <1m, 1m, 2m, 3m, 6m, 12m | Maternal vaccination | No intervention | CEA, CUA |
| Ishiwada N, 2024 | Japan | Healthcare payer | Pregnant women | ≤27 wGA, 28–31 wGA, 32–36 wGA, ≥37 wGA | Combined of RSVpreF and palivizumab | Palivizumab | CEA, CUA |
| Shoukat A, 2023 | Canada | Healthcare and societal. | Pregnant women | <29 wGA, 29–32 wGA, 33–36 wGA, 37+ wGA | Nirsevimab | RSVpreF | CEA, CUA |
| Mizukami A, 2024 | Japan | Public healthcare payer | ≥60y | 60, 65, 75 | RSVPreF3 OA | No intervention | CEA, CUA |
| Averin A, 2025 | Germany | Societal | ≥60y | 60, 70, 80 | RSVpreF vaccination | No intervention | CEA, CUA |
| Gourzoulidis G, 2024 | Greece | Public payer’s | ≥60y | 60y, 65y, 75y, 85y | RSVpreF vaccination | No intervention | CEA, CUA |
| Rey-Ares L, 2024 | Argentina | Healthcare system | Pregnant women | 0-11m | Maternal RSVpreF | No intervention | CEA |
| Laufer RS, 2021 | Mali | Government, donor, and societal. | Pregnant women | 1m, 2m, 3m, 4m, 5m, 6m | (1) Licensed mAb;  (2) Long-acting mAb;  (3) Maternal vaccination | No intervention | CEA, CUA |
| Hutton DW, 2024 | US | Societal | Pregnant women | 0-23m | Maternal RSVpreF | No intervention | CEA |
| Bugden S, 2025 | Canada | Healthcare and societal | Infants | 0-11m | (1) Palivizumab;  (2) Nirsevimab | No intervention | CEA |
| Meijboom MJ, 2012 | Netherlands | Societal | Infant |  | 3-dose RSV vaccination | No intervention | CEA, CUA |
| Moghadas SM, 2024 | United States | Societal | Older Adults | 60y, 65y, 70y, 75y, 80y, 85y | (1) Arexvy;  (2) Abrysvo;  (3) Arexvy and Abrysvo | No intervention | CEA, CUA |
| Li X, 2022 | Norwegian |  | Pregnant and Children |  | (1) Maternal vaccine;  (2) mAb | No intervention | CEA, CUA |
| Li X, 2023 |  | Healthcare payer and societal | Children |  | (1) Maternal vaccine;  (2) mAb | No intervention | CEA, CUA |
| Rudd M, 2025 | Canada | Health system | Older adults | PHAC: 50y, 60y, 65y, 70y, 75y, 80y GSK: 50y, 60y, 65y, 70y, 75y, 80y Pfizer: 18-49y, 50y, 60y, 70y, 80y | Three static cost-utility models |  | CEA, CUA |
| Gebretekle GB, 2024 | Canada | Health system and societal | Canadian infants | <33 wGA, 33^0^-36^6^ wGA, >37 wGA | (1) Year-round RSVpreF for all pregnant people (2) Year-round RSVpreF plus nirsevimab for high-risk infants (3) Seasonal nirsevimab for all infants with catch-up (4) Seasonal nirsevimab for all infants without catch-up (5) Year-round nirsevimab for all infants | Palivizumab for infants at high risk | CEA, CUA |
| Liu D, 2021 | China | Societal | Pediatric |  | (1) Maternal immunisation;  (2) Infant mAb;  (3) Pediatric immunisation;  (4) Their combinations | No intervention | CEA, CUA |
| La EM, 2024 | United States | Societal | ≥60 y |  | RSVPreF3 | No intervention | CEA, CUA |
| Hutton DW, 2024 | US | Societal | ≥60 y | 60y, 65y, 70y, 75y, 80y | (1) Arexvy;  (2) Abrysvo | No intervention | CEA, CUA |
| Huerta JL, 2025 | Mexico | Healthcare system | Pregnant women |  | Maternal vaccination | No intervention | CEA, CUA |
| Nourbakhsh S, 2021 | Canada |  | Infant and pregnant | Infants0-2m, 3-5m, 6-11m Children: 12-23m, 24-35m, 36-47m, 5-18y Adults: ＞18 y | (1) Long-acting mAb; (2) Palivizumab;  (3) Maternal vaccines | No intervention | CEA, CUA |
| NazareNo AL, 2025 | Australia | Healthcare system | All age groups | 0y, 1y, 2y, 3y, 4y, 5y, 15y, 25y, 35y, 45y, 55y, 65y, 75y | Maternal vaccination | No intervention | CEA, CUA |
| Koltai M, 2023 | Kenya and South Africa |  | Pregnant and children under 5 years |  | (1) Maternal vaccination; (2) mAb | No intervention | CEA |
| Hodgson D, 2020 | UK |  | Infants and pregnant | Monthly up to 11 months, and 1, 2, 3, 4, 5–9, 10–14, 15–24, 25–34, 35–44, 45–54, 55–64, 65–74, 75+years | (1) Long-acting mAb;  (2) Infants vaccination;  (3) Maternal vaccination | Palivizumab | CEA, CUA |
| Guiñazú G, 2024 | Argentina | Societal | Infants |  | (1) Long-acting mAb;  (2) Maternal vaccine | No intervention | CEA |
| Zeevat F, 2022 | Netherlands and UK | Netherlands: Societal  UK: Third-party payer | Netherlands: ≥60 y UK: ≥65 y | Netherlands: 60y, 65y, 75y, 85y UK: 65y, 75y | RSV vaccine | No intervention | CEA, CUA |
| Li X, 2020 | Gavi-eligible countries | Health care payer | from birth until 5 years of age |  | (1) Maternal vaccination; (2) mAb | No intervention | CEA |
| Shoukat A, 2024 | Canada | Healthcare and societal | ≥ 60y | 60y, 65y, 70y, 75y, 80y, 85y | (1) Arexvy;  (2) Abrysvo | No intervention | CEA, CUA |
| Baral R, 2020 | 131 LMICs | Health system | Infants and pregnant |  | (1) Maternal vaccination; (2) mAb | No intervention | CEA |
| Do LAH, 2023 | Vietnam | Societal | Infants |  | (1) RSVpreF vaccine;  (2) Nirsevimab | No intervention | CEA |
| Laufer RS, 2023 | Mali | Societal, government, donor | Infants |  | (1) mAb;  (2) Maternal RSV vaccine;  (3) Pediatric vaccine(10/14weeks);  (4) Pediatric vaccine(6/7months) | No intervention | CEA |
| Rietveld E, 2010 | Netherlands | Societal | Children |  | Palivizumab | No intervention | CEA |
| Pouwels KB, 2016 | Turkey | Societal | Infants |  | (1) Vaccinating infants at 2 and 4 months of age;  (2) Vaccinating pregnant women;  (3) Vaccinating pregnant women and infants at 2 and 4 months of age | No intervention | CEA |
| Postma MJ, 2023 | Belgium | Healthcare payer | ≥ 60y |  | (1) Vaccine protection durations 1 year;  (2) Vaccine protection durations 3 years;  (3) Vaccine protection durations 5 years | No intervention | CBA |
| Hodgson D, 2024 | UK | National Health Service | Pregnant women at 24–36 weeks gestation; infants < 6m |  | Long-acting mAb | Maternal vaccination | CEA, CUA |
| Tuite AR, 2024 | Canada | Health system, a societal | Adults ≥60 Years | 50y, 60y, 65y, 70y, 75y, 80y | (1) RSV PreF3;  (2) RSV PreF | No intervention | CEA, CUA |
| Meijboom MJ, 2013 | Netherlands |  | Adults ≥60 Years | 60y, 65y, 75y, 85y | (1) Vaccine effectiveness of 100% (2) Vaccine effectiveness of 40% | No intervention | CEA, CUA |
| Gessner BD, 2000 | United States | Societal | Adults ≥65 Years |  | (1) Without a 10% vaccine effectiveness against COPD; (2) With a 10% vaccine effectiveness against COPD | No intervention | CEA, CUA |

m: month; y: year; CBA: cost-benefit analysis; CEA: cost-effectiveness analysis; CUA: cost-utility analysis; COPD: chronic obstructive pulmonary disease; mAb: monoclonal antibodies; wGA: weeks gestational age;

**Table S3.** Core model characteristics

| Study | Model type | Model status | Time horizon | Circle | Discount rate of efficacy | Discount rate of cost | Efficacy indication | Costs |
| --- | --- | --- | --- | --- | --- | --- | --- | --- |
| Thomas G, 2015 |  |  | 5 years |  |  | 3.50% | NA | (1) Direct costs: the individual patient medical care in hospital as well as the drug and administration with non-individual outlays on such items as research, avoidance and assessment. (2) Indirect costs: society’s evaluation of the loss of life and the reduced quality of health that results from RSV illnesses. |
| Cromer D, 2017 | Decision tree | Symptomatic RSV, no Symptomatic RSV, no health care use, GP consultation, hospitalisation, intensive care admission, death |  |  | 3.50% | 3.50% | QALY | Direct costs: GP visit, hospital admission, ventilation or intensive care admission |
| Wang Y, 2023 | Decision tree | Severe vaccine-associated adverse events, RSV infection, RSV-associated LRTD, RSV-associated ARI, hospitalization, outpatient care, Self-managed care, high-risk group, death | 2 years |  | 3% | 3% | QALY | Direct medical costs: RSV vaccines, treatment(self-managed care, outpatient care, and hospitalization) for RSV-associated LRTD/ARI, and vaccine-related severe adverse events. |
| Álvarez Aldean J, 2024 | Markov | RSV-infected, hospitalizations, ED visits, primary care visits, deaths | 1 year | 1 month | 3% | 3% | QALY | Direct medical costs: cost of Maternal vaccine and Administration, cost of RSV hospitalization, cost of RSV ED encounter, cost of RSV PC encounter |
| Ishiwada N, 2024 | Markov | Medically attended RSV, hospitalization, emergency visit, OV, recovery, death | Lifetime |  | 2% | 2% | QALY | (1) Direct costs: costs of intervention (vaccine/drug costs with administration costs) and medical care. (2) Productivity losses: the caregivers’ absence from work due to providing care to infected infants, lost labor opportunities that might have been gained after maturity of the children who died of RSV infection, and absence from work of pregnant women receiving maternal vaccination at a hospital visit and caregivers taking children to hospital for palivizumab administration |
| Shoukat A, 2023 | Discrete-event simulation | Symptomatic RSV, medically attended, outpatient, inpatient, ICU, recovered, death | 1 year |  | 1.50% | 1.50% | QALY | (1) Direct costs: office visit, ED visit, hospitalisation, as well as 30 days’ follow up for hospitalized infants (2) Indirect costs: out-of-pocket expenses, loss of productivity by parents, and monetary loss of life due to RSV related infant mortality. |
| Mizukami A, 2024 | Markov | RSV-ARI, RSV-LRTD, RSV-URTD, post-RSV, reinfection with RSV, reinfection RSV-LRTD, reinfection RSV-URTD, death | 3 years | 1 month | 2% | 2% | QALY | (1) Direct medical costs (2) Vaccine-related costs: the vaccine price, the administration cost, the cost per adverse event induced by vaccination |
| Averin A, 2025 | Markov | Hospital, outpatient, RSV-related deaths | 39 years | 1 month | 3% | 3% | QALY | (1) Direct costs: Setting-specific direct medical costs of RSV-LRTD, vaccine costs, vaccine administration costs (2) Indirect costs: patient productivity loss were estimated based on labor force participation, average daily wage, and work-loss days due to RSV-related illness. |
| Gourzoulidis G, 2024 | Markov | RSV-Hospital, RSV-ED, RSV-OV, Death | Lifetime | 1 month | 3.50% | 3.50% | QALY | Direct costs: vaccination and RSV-attributable direct medical costs estimated for episodes of RSV-Hospital, RSV-ED, and RSV-OV. |
| Rey-Ares L, 2024 | Markov | RSV-LTRI (hospital, ED, PO), deaths |  |  | 3% | 3% | QALY | (1) Direct medical costs: cost of vaccine and cost of vaccine administration (2) Indirect costs: patient productivity loss were estimated based on labor force participation, average daily wage, and work-loss days due to RSV-related illness |
| Laufer RS, 2021 | Markov | RSV-LTRI, inpatient care, outpatient care, appropriate level of care not provided, survival, death | 6 months |  | 3% | 3% | DALY | Inpatient care costs, outpatient care costs, intervention delivery cost per dose |
| Hutton DW, 2024 |  | OVs, ED (ED) visits, hospitalizations, premature deaths | first RSV season | 1 month | 3% | 3% | QALY | The cost of RSVpreF, cost to administer the vaccine, costs of palivizumab for high-risk infants, productivity losses from RSV disease and adverse events, RSV health care utilization costs, caregiver productivity losses and infant productivity losses, overall general health care costs including those not related to RSV |
| Bugden S, 2025 | Decision tree | Medically-attended RSV, primary care visit, ED visit, hospitalisation, ICU admission, GW, death, survive | 1 year |  |  |  | QALY | Direct health care costs |
| Meijboom MJ, 2012 | Markov | Symptomatic RSV infection, acquired immunity, health care utilization, death, vaccinated |  |  | 1.50% | 4% | QALY | Costs of GP-visits and costs of hospitalization, cost of lost workday |
| Moghadas SM, 2024 | Discrete-event simulation | Symptomatic RSV, medically attended, outpatient, Inpatient, office visit, ED visit, GW, ICU, mechanical ventilation, recovered, death |  |  | 3% | 3% | QALY | (1) Direct costs: office visits, ED visits, and hospitalization (2) Indirect costs: productivity loss was calculated for the duration of RSV disease and associated outcomes, remaining productivity life expectancy |
| Li X, 2022 | MCMARCEL | Symptomatic RSV, no symptomatic RSV, death |  |  | 4% | 4% | QALY | Year-round mAb and MI programs cost, delivery cost, the seasonal mAb program cost |
| Li X, 2023 | 3 static models and 2 dynamic models |  | 1 year; 10 year |  | 3% | 3% | QALY | Cost per hospitalisation, Cost per hospital OV, Cost per primary care visit, ICU admission, Length of stay in hospital or ICU, Transportation for parents per hospitalisation or ICU admission, Cost of productivity loss, Workdays lost due to RSV, Single-dose maternal vaccine (MV) infected child, Single-dose monoclonal antibody (mAb), Delivery cost per dose, Programme implementation costs (one-off) |
| Rudd M, 2025 | Decision tree |  | 3-year |  | 1.50% | 1.50% | QALY | vaccination costs and healthcare costs. |
| Gebretekle GB, 2024 | Decision tree | Healthy, RSV-infected, death |  |  | 1.50% | 1.50% | QALY | Costs included immunization costs and RSV-associated medical expenses for outpatient healthcare provider visits, ED visits, hospitalization in a pediatric GW, and ICU |
| Liu D, 2021 | Decision tree | RSV-outpatient, RSV-inpatient, RSV-death. |  |  | 3% | 3% | QALY | Direct medical costs, direct non-medical costs, and indirect costs |
| La EM, 2024 | Markov | LRTD, URTD, ARI, post RSV, death | 5-year | 1-month | 3% | 3% | QALY | (1) Direct costs associated with healthcare resource use and medications;  (2) Indirect costs due to productivity losses, vaccine acquisition and administration costs |
| Hutton DW, 2024 | Markov |  | Lifetime |  | 3% | 3% | QALY, LY | (1) Direct medical costs: costs of vaccination, RSV disease, and all other healthcare;  (2) Indirect medical costs: productivity costs |
| Huerta JL, 2025 | Markov |  | Lifetime |  | 5% | 5% | QALY, LY | (1) Direct Costs: hospitalization-specific cost, ED-specific costs, costs associated with intervention (vaccine and administration costs), medical care (2) Indirect Costs: caregiver lost productivity and travel expenses, productivity losses |
| Nourbakhsh S, 2021 | Discrete-event simulation |  | 1-year |  |  |  | QALY | OV, pediatric ward, ICU |
| NazareNo AL, 2025 | Dynamic model | Symptomatic RSV, asymptomatic RSV, death, survival | 1-year |  |  | 5% | QALY | Direct healthcare costs: GP visit, ED visit, and hospitalisation (non-ICU and ICU), cost of the vaccine and its administration |
| Koltai M, 2023 | McMarcel | ARI, SARI, death |  |  |  |  | DALY | South Africa: cost of outpatient care and the cost of hospitalisation by age group Kenya: direct out-of-pocket payments for drugs and transportation and loss of income due to absence from work |
| Hodgson D, 2020 | transmission dynamics |  | 10-year |  | 3.50% | 3.50% | QALY | Costs per GP visit, per hospital bed day, administration of prophylactics (per course) |
| Guiñazú G, 2024 | UNIVAC | RSV (non-severe), RSV (severe) cases | 10 years |  | 3% | 3% | DALY | (1) Direct medical costs: drugs, diagnostics;  (2) Direct nonmedical costs: transportation;  (3) Indirect costs: opportunity costs of lost income |
| Zeevat F, 2022 | Decision tree |  | 1year |  |  |  | QALY | (1) Direct; (2) Indirect costs: productivity losses |
| Li X, 2020 | A multi-country RSV impact model | Symptomatic RSV; no symptomatic RSV; deaths from other causes |  |  | 3% | 3% | DALY | Direct health care costs |
| Shoukat A, 2024 | Discrete-event simulation | Symptomatic RSV; outpatient; inpatient | two RSV seasons |  | 1.50% | 1.50% | QALY | (1) Direct costs: physician visits, ED visits, hospitalisation, and hospital overhead;  (2) Indirect costs: loss of productivity |
| Baral R, 2020 |  |  |  |  | 3% | 3% | DALY | Intervention cost; intervention delivery costs, treatment cost |
| Do LAH, 2023 | UNIVAC |  | 10 years |  | 3% | 3% | DALY | Direct costs: price per dose, syringe price per dose, safety box price per dose, international handling, international delivery, incremental health system cost per dose |
| Laufer RS, 2023 | Decision tree | RSV-LRTD (impatient care, outpatient care, appropriate level of care not provided), no RSV-LRTD |  |  | 3% | 3% | DALY | Medical treatment and hospitalization costs, associated procurement, storage, and administration costs |
| Numa A, 2000 |  |  |  |  |  |  | hospital days saved | Hospital bed costs, Capital (infrastructure) and recurrent expenses |
| Pouwels KB, 2016 | Markov | Susceptible, RSV infection, RSV Death, vaccine immune, natural immune, death | 1 month |  | 3% | 3% | QALY | Cost of GP visit, cost of RSV-hospitalization, productivity loss per GP visit, Productivity loss per hospitalization, cost of lost workday, price of vaccine per dose |
| Postma MJ, 2023 | Markov | Symptomatic RSV (inpatient, outpatient, non-medically attended), no symptomatic RSV, |  |  | 3% | 3% | NA | Patient productivity loss, cost of caregiver productivity loss |
| Hodgson D, 2024 | Dynamic transmission |  | 10 years |  | 3.50% | 3.50% | QALY | GP consultation, A&E attendance, hospital admission episode, ICU admission episode |
| Tuite AR, 2024 | individual-based | Health care provider visit; ED visit; hospital admission; hospital admission with ICU admission; recovered, death | 3 years |  | 1.50% | 1.50% | QALY | Patient productivity loss, caregiver productivity loss, and out-of-pocket medical costs. |
| Meijboom MJ, 2013 |  |  |  |  | 1.50% | 4% | QALY | Costs due to general practice visits, hospitalization, antibiotic prescriptions and vaccination. |
| Gessner BD, 2000 | Decision tree |  | 1years |  | 3% | 3% | QALY | Physician OV, hospitalization for RSV pneumonia; Hospitalization for COPD, yearly nursing home costs, average future yearly medical costs of persons aged 65±74; average future yearly medical costs of persons age 75±82; outpatient medications for RSV infection |

NA: not applicable; ED: emergency department; PO: physician’s office; OV: outpatient visit; URTD: upper respiratory tract diseases; LRTD: lower respiratory tract diseases; ARI: acute respiratory infection; GW: general ward; GP: General Practitioner

**Table S4.** Detailed base case results of the economic evaluation of RSV preventive strategies

| study | Incremental costs | Benefit-cost ratio | Incremental effectiveness | ICER | Base case results |
| --- | --- | --- | --- | --- | --- |
| Thomas G, 2015 | £14263717.08 | 1.3906 |  |  | Passive immunisation programme was a cost-saving choice. |
| Cromer D, 2017 |  |  |  |  | The MCEP for combination of a newborn and infant programme was £246 (95% UI 219–275). |
| Wang Y, 2023 | ABRYSVO® vs. No Vaccination: 87 USD AREXVY® vs. No Vaccination: 123 USD |  | ABRYSVO® vs. No Vaccination: 0.000647 QALY AREXVY® vs. No Vaccination: 0.000568 QALY | ABRYSVO® vs. No Vaccination: 137, 907 USD/QALY AREXVY® vs. No Vaccination: 219, 299 USD/QALY | ABRYSVO® group was accepted as the cost-effective option at the 25% US vaccine price level. |
| Álvarez Aldean J, 2024 | €-1.80 million |  | 551 QALYs | Dominant | Maternal vaccination resulted in a dominant strategy compared to no intervention in the Spanish NHS setting. |
| Ishiwada N, 2024 | Payer perspective: ¥2141.4 Societal perspective: ¥1987.1 |  | 428.4 QALYs | Payer perspective: ¥4, 998, 847/QALY Societal perspective: ¥4, 638, 509/QALY | A combination prophylaxis was cost-effective under the ICER threshold of ¥5 million per QALY. |
| Shoukat A, 2023 | Birth cohorts: CAD$ 467 Pregnant women: CAD$ 4501 |  | Birth cohorts: 0.111 QALY Pregnant women: 0.109 QALY | Birth cohorts: CAD$ 4200 Pregnant women: CAD$ 41, 321 | Nirsevimab would be cost-effective from a societal perspective for a PPD of up to $290. |
| Mizukami A, 2024 | JPY 456.13 billion |  | 109119QALY | 4180084 JPY/QALY | Vaccination was cost-effective compared to no intervention. |
| Averin A, 2025 | 1.8 € billion |  | 49,576 QALYs | 36,064 €/QALY | RSVpreF has the potential to greatly reduce the public health and economic burden of RSV among older adults in Germany |
| Gourzoulidis G, 2024 | EUR 142, 59 |  | 7,230 QALY;  18,118 hospitalized RSV cases avoided | EUR 19,723/QALY;  EUR 7,870 per hospitalized RSV case avoided | RSVpreF was cost-effective in Greek adults over 60 years of age. |
| Rey-Ares L, 2024 | $-6.72 million |  | 2061QALY |  | The price of RSVpreF was estimated to be $74.46 per dose |
| Laufer RS, 2021 | Short-acting mAb: USD 5, 032, 307 (Societal perspective) Long-acting mAb: USD 1, 714, 231 (Societal perspective) Maternal vaccine: USD 1, 178, 325 (Societal perspective) |  | Short-acting mAb: 1206 DALY Long-acting mAb: 1059 DALY Maternal vaccine: 146 DALY | Short-acting mAb: USD 4164/DALY (Societal perspective) Long-acting mAb: USD 1614/DALY (Societal perspective) Maternal vaccine: USD 8038/DALY (Societal perspective) | Long-acting monoclonal antibody is likely to be cost-effective from both the government and donor perspectives at $3 per dose. |
| Hutton DW, 2024 | $ 513 million |  | 1, 294QALY | $396, 280/QALY | RSVpreF has the potential to be cost-effective in specific circumstances, particularly when administered at the ideal gestational and seasonal time. |
| Bugden S, 2025 |  |  |  | (Per one averted hospitalisation)  NIRS HR: Cost saving NIRS HR + MR: $8139 ABR SEASONAL: $23, 896 ABR SEASONAL + NIRS: $23, 790 ABR ALL: $36, 396 ABR ALL + NIRS: $36, 378 NIRS < 6: $49, 683 NIRS ALL: $53, 113 | Nirsevimab administered only to high-risk infants is the most cost-effective strategy compared to palivizumab in southern Canada. |
| Meijboom MJ, 2012 | 15.6 million€ |  | 397QALYs | 34143€/QALY | Vaccination of infants against RSV might be cost-effective. |
| Moghadas SM, 2024 | ①Arexy only vs No vaccination: 5562363$ ②Abrysvo only vs No vaccination: 5368121$ ③Arexy and Abrysvo vs No vaccination: 5440866$ |  | ①Arexy only vs No vaccination: 59.19QALYs ②Abrysvo only vs No vaccination: 56.71QALYs ③Arexy and Abrysvo vs No vaccination: 57.74QALYs | ①Arexy only vs No vaccination: 93981$/QALY ②Abrysvo only vs No vaccination: 94651$/QALY ③Arexy and Abrysvo vs No vaccination: 94234$/QALY | Vaccination programs could be cost-effective for a PPD up to $127 with Arexvy and $118 with Abrysvo over the first RSV season under the WTP of $95 000 per QALY gained. |
| Li X, 2022 |  |  |  |  | At NOK 500 per dose, either mAb “Nov-Feb,” “Oct-Feb,” or “Oct-Mar” is the cost-effective intervention. |
| Li X, 2023 | MV:  UA: 1652384€ NV: 1563621€ SPS: 1548846€ SPD: 21593498€ LSHTM: 17761583€ mAb:  UA: 283229€ NV: 50655€ SPS: 40118€ SPD: 5589180€ LSHTM: 15719086€ |  | MV:  UA: 5QALYs NV: 4QALYs SPS: 5QALYs SPD: 11QALYs LSHTM: 109QALYs mAb:  UA: 24QALYs NV: 21QALYs SPS: 25QALYs SPD: 163QALYs LSHTM: 447QALYs | payers perspective MV:  UA: 402349€/QALY NV: 463979€/QALY SPS: 366437€/QALY SPD: 1973816€/QALY LSHTM: 178322€/QALY mAb:  UA: 71522€/QALY NV: 69419€/QALY SPS: 61626€/QALY SPD: 101282€/QALY LSHTM: 54272€/QALY societal perspective MV:  UA: 332952€/QALY NV: 375702€/QALY SPS: 297665€/QALY SPD: 1901299€/QALY LSHTM: 162266€/QALY mAb:  UA: 11658€/QALY NV: Dominated SPS: 1635€/QALY SPD: 34327€/QALY LSHTM: 35205€/QALY | The LSHTM model had the highest QALY losses due to RSV episodes because it attributed QALY losses to non-MA symptomatic RSV infections. The SPD model reported the lowest QALY losses due to RSV episodes because it estimated lower incidences for both MA and non-MA symptomatic infections. |
| Rudd M, 2025 |  |  |  |  | Vaccination for people with CMCs over the age of 70 years was the optimal cost-effectiveness policy when using a threshold of $50,000/QALY. |
| Gebretekle GB, 2024 |  |  |  | Seasonal nirsevimab for infants at moderate or high risk, no catch-up vs Standard of care (palivizumab for infants at high risk): Dominated Seasonal nirsevimab for infants at moderate or high risk, with catch-up: 27891$/QALY Year-round nirsevimab for infants at moderate or high risk: Dominated Year-round RSVpreF plus nirsevimab for infants at high-risk: 204621$/QALY Year-round RSVpreF for all pregnant women and pregnant people: Dominated Seasonal nirsevimab for all infants, No catch-up: Dominated Seasonal nirsevimab for all infants, with catch-up: 512265$/QALY Year-round nirsevimab for all infants: Dominated | Seasonal nirsevimab for infants at moderate- and high-risk with catch-up was the most cost-effective strategy with an ICER of $27, 891 per QALY when compared to palivizumab. |
| Liu D, 2021 |  |  |  |  | Year-round infant mAb plus paediatric immunisation is the most cost-effective among all the year-round strategies |
| La EM, 2024 | $4, 504, 734, 996 |  | 244424QALYs | 18430$/QALY | Adjuvanted RSVPreF3 vaccination is a cost-effective option for the prevention of RSV in US adults aged ≥ 60 years. |
| Hutton DW, 2024 | vaccine vs No vaccine ($) GSK: ①60-65: 1110②65-70: 926③70-75: 709④75-80: 421⑤≥80: 497 Pfizer: ①60-65: 1153②65-70: 951③70-75: 736④75-80: 430⑤≥80: 511 |  | vaccine vs no vaccine GSK: ①60-65: 2877②65-70: 3600③70-75: 3035④75-80: 4549⑤≥80: 4484QALYs Pfizer: ①60-65: 3477②65-70: 4216③70-75: 3545④75-80: 5110⑤≥80: 5078QALYs | vaccine vs no vaccine($/QALY) GSK: ①60-65: 385829②65-70: 253967③70-75: 233472④75-80: 92438⑤≥80: 110830 Pfizer: ①60-65: 331486②65-70: 225521③70-75: 207453④75-80: 84652⑤≥80: 100726 | Vaccination in adults aged ≥60 years may be cost-effective, particularly in those of more advanced age. |
| Huerta JL, 2025 | Maternal vaccine vs no intervention: $905.84millon |  | Maternal vaccine vs no intervention: 3493QALYs | Maternal vaccine vs no intervention: $247102million/QALY | Year-round RSVpreF maternal vaccination would likely be a cost-effective program |
| Nourbakhsh S, 2021 | Palivizumab vs no intervention: 84750$ Long-acting mAb vs no intervention: -71927$ Maternal vaccine vs no intervention: 14502$ Maternal vaccine + LAMA for preterm and chronically ill infants vs no intervention: -25785$ Palivizumab for healthy infants vs Palivizumab: 392677$ LAMA for healthy infants vs LAMA: 35084$ |  | Palivizumab vs no intervention: 0.084QALYs Long-acting mAb vs no intervention: 0.0814QALYs Maternal vaccine vs no intervention: 0.0639QALYs Maternal vaccine + LAMA for preterm and chronically ill infants vs no intervention: 0.1258QALYs Palivizumab for healthy infants vs Palivizumab: 0.8905QALYs LAMA for healthy infants vs LAMA: 0.8906QALYs | Palivizumab vs no intervention: 1011139$/QALY Long-acting mAb vs no intervention: 883539$/QALY Maternal vaccine vs no intervention: 227286$/QALY Maternal vaccine + LAMA for preterm and chronically ill infants vs no intervention: 204621$/QALY Palivizumab for healthy infants vs Palivizumab: 441023$/QALY LAMA for healthy infants vs LAMA: 39414$/QALY | Palivizumab offered to full-term infants aged 0-2 months and high-risk for complicated RSV disease is not cost-effective, compared to preterm/chronically ill infants under 1 year of age. |
| NazareNo AL, 2025 | Maternal vaccination vs no vaccination: 2918290.81$AU |  | Maternal vaccination vs no vaccination: 255.92QALYs | Maternal vaccination vs no vaccination: 11403.10$AU/QALY | Maternal vaccination can be cost-effective up to $AU 120 per dose compared to no vaccination at WTP of $AU 50,000 per QALY gained. |
| Koltai M, 2023 |  |  |  |  | Interventions against RSV disease may be more cost-effective than previously estimated. |
| Hodgson D, 2020 |  |  |  |  | Long-acting monoclonal antibody programme was cost-effective. |
| Guiñazú G, 2024 | mAb: $ 204,933, 25; Maternal RSV vaccine: $193,553,122 |  |  |  | Long-acting monoclonal antibody and maternal RSV vaccine are both cost-effective compared to no intervention. |
| Zeevat F, 2022 |  |  |  |  | Justifiable vaccine prices of €16.38 and €50.03 were found, based on applying the lower and higher WTP thresholds, respectively. |
| Li X, 2020 | mAb: 430531000 USD; maternal RSV vaccine: 220775 USD |  | mAb: 137000 DALY; maternal RSV vaccine: 98000 DALY |  | The maternal strategy is the most cost-effective strategy in LMICs (1000–8000 USD per DALY). |
| Shoukat A, 2024 | $403, 800 |  | 8.13QALY | $49653/QALY | Arexvy would be cost-effective from a societal perspective when vaccinated to 90% of residents in LTCHs for a PPD up to $163 at a WTP of $50, 000 per QALY gained. |
| Baral R, 2020 | $54.636 bilian |  | 3730000 DALY | $1342/DALY | Maternal vaccine and mAbs were cost-effective in 60 and 118 countries at a 50% gross domestic product per capita threshold, respectively. |
| Do LAH, 2023 | $213,022,691 |  | 61882 DALY | $3442/DALY | Both RSVpreF and Nirsevimab have the potential to be cost-effective. |
| Laufer RS, 2023 | $523, 851 |  | 878 DALY | $597/DALY | Extended half-life RSV mAbs would be impactful and efficient components of prevention strategies in LMICs such as Mali. |
| Pouwels KB, 2016 | 11.2 billion TL |  | 2172 QALY | 51969TL/QALY | All strategies remained slightly below the threshold of 3 times the GDP per capita. |
| Hodgson D, 2024 | £118731529 |  | 147 QALY |  | LAMA could be cost-effective for up to £84 CCPA and MV could be cost-effective for up to £80 CCPA. |
| Tuite AR, 2024 |  |  |  |  | Vaccinating adults aged 70 years and older with 1 or more chronic medical condition was the optimal strategy for a cost effectiveness threshold of $50 000 per QALY. |
| Meijboom MJ, 2013 |  |  |  | €133068/QALY | Vaccination of the complete 60+ elderly cohort would not be cost-effective. |
| Gessner BD, 2000 | $10, 037, 000 |  | 1879 QALY | $5342/QALY | RSV vaccine would be cost-effective for the elderly population, with cost-effectiveness ratios similar to those for influenza vaccine. |

RSV: respiratory syncytial virus; ICER: incremental cost effectiveness ratio; QALY: quality-adjusted life years; DALY: disability adjusted life years; WTP: willingness-to-pay; LAMA: long-acting monoclonal antibodies; mAb: monoclonal antibodies; MA: medically attended; PPD: price-per-dose; CCPA: could be cost-effective for purchasing and administration; NHS: national healthcare system

**Table S5.** Results of sensitive analysis of the economic evaluation of RSV preventive strategies

| Study | Sensitive analysis | DSA results | PSA results | Scenario analysis |
| --- | --- | --- | --- | --- |
| Thomas G,2015 | DSA | Prices of the drug |  |  |
| Cromer D,2017 | DSA | Vaccine efficacy, and for the maternal or newborn strategy, to the duration of vaccine protection |  |  |
| Wang Y,2023 | DSA PSA | VE against RSV-ARI in season 2, multiplier for under-detection of RSV by rapid antigen assays, RSV attack rate (0.007-0.0721) | The ICERs of the ABRYSVO® group (versus no vaccination) were below the WTP threshold in 97.66%, 15.77%, 0.07%, and 0% of the time at 25%, 50%, 75%, and 100% US vaccine price, respectively. The ICERs of the AREXVY® group against no vaccination were below the WTP threshold in 53%, 0.05%, 0%, and 0% of simulations at 25%, 50%, 75%, and 100% US vaccine price levels, correspondingly. | 25%, 50%, 75%, and 100% US vaccine price levels |
| Álvarez Aldean J,2024 | DSA PSA | The only variations to the parameters that affected the outcomes were the 25% decrease in the effectiveness of maternal vaccine, in the incidence of RSV hospitalization and in the cost of hospitalizations, and the 25% increase in the cost of maternal vac‑ cine. | a total of 1000 simulations, showed that 99% of iterations fell below the WTP threshold of €25,000/QAL, meaning maternal immunization was cost-effective in 99% of iterations. | Reducing vaccination coverage from 70 to 50% |
| Ishiwada N,2024 | DSA | cost of RSVpreF vaccine, cost of palivizumab, effectiveness of RSVpreF vaccine |  | In scenario 2, premature (≤ 31 wGA) regardless risk in infants were eligible to receive palivizumab. The weeks of pregnancy at vaccination (scenario 7), the prescription time of palivizumab per year (scenario 9), the use of palivizumab in the combination prophylaxis (scenario 10), RSV incidence (scenario 11), and productivity loss for visiting hospital for vaccination or palivizumab prescription (scenario 16) were impactful scenarios on ICER. |
| Shoukat A,2023 | PSA |  | The probabilities of these programs being cost-effective at their maximum PPD were 52%, 51%, 83%, and 55% in L1, L2, L3, and L4, respectively. MI was cost-effective for a PPD up to $200, with the probability of 87%. | (i) preterm infants ≤32 wGA and infants with CLD or CHD condition (L1); (ii) preterm infants ≤36 wGA and infants with CLD or CHD condition (L2); (iii) preterm infants (≤36 wGA), infants with CLD or CHD, and term infants born during RSV season (L3); and (iv) the birth cohort (L4) |
| Mizukami A,2024 | DSA PSA | Assumptions regarding RSV incidence; waning rates against RSV-LRTD; and peak vaccine efficacy against RSV-LRTD | From the PSA, results were found to be robust, with around 73.0% and 84.0% of simulations resulting in an ICER below WTP thresholds of JPY 5,000,000 and JPY 6,000,000, respectively | In scenario analyses, the cost-effectiveness of alternative vaccination target groups was explored: ≥65 years, ≥70 years, and ≥75 years. A scenario analysis explored outcomes over a two-year time horizon, instead of the base case three-year horizon. |
| Averin A,2025 | DSA PSA | RS Hospital incidence rates, vaccine effectiveness, case fatality rates, utility values | All PSA replications were in the northeast quadrant of the scatterplot and 92.5% were below 50,000 €/QALY and 100% were below 100,000 €/QALY. The mean ICER in PSA was 36,518 €/QALY | scenario analyses – considering alternative waning profiles, utility values, discount rates, and hospitalization costs, respectively – were conducted |
| Gourzoulidis G,2024 | DSA PSA | RSVpreF unit cost and RSV rate related to hospitals | At the threshold of EUR 44,000, vaccination with RSVpreF had a 96% probability of being a cost-effective strategy versus the no vaccination strategy. | (1) adults aged 60 and older at high risk, (2) adults aged 65 and older at high risk, (3) adults aged 75 and older at high risk, (4) adults aged 60–74 at high risk, (5) all adults aged 65 and older, and (6) all adults aged 75 and older. |
| Rey-Ares L,2024 | DSA PSA | Vaccine effectiveness, Incidence rates, Direct costs | Probabilistic Sensitivity Analyses Results from probabilistic sensitivity analyses, suggest that RSVpreF would remain cost effective at prices up to $75.02 (95% CI 54.24-97.30) | The scenario analysis considered shorter (6 months) and longer (12 months) duration of protection |
| Laufer RS,2021 | DSA |  |  |  |
| Hutton DW,2024 | DSA PSA | Probability of Prematurity Due to Vaccination, RSV QALYS Lost, RSVpreF Vaccine Cost/Dose | 53% had a cost <$500 000 per QALY saved | We conducted several scenario analyses with varying efficacy assumptions. We also evaluated a combination of assumptions on efficacy, hospitalization costs, and mortality risks. |
| Bugden S,2025 | DSA PSA | In southern Canada, the most cost-effective strategy is impacted only by changing product prices. In the northern regions, the optimal strategy also depends on hospitalisation rates. | In southern Canada and Nunavut, the base case optimal strategy is the most cost-effective strategy in 85% and 68% of iterations at a WTP threshold of $100,000 per QALY, | First, we dropped the base case assumption of constant product efficacy and permitted the efficacies of nirsevimab, RSVpreF, and palivizumab to wane over the course of the year. Second, we ran the analyses from a societal perspective, incorporating out-of-pocket costs and lost wages for families with hospitalized infants. Third, we ran the analyses from an expanded societal perspective with the inclusion of monetary productivity cost and QALY loss associated with infant mortality. |
| Meijboom MJ, 2012 | DSA PSA | Incidence rates of RSV-related GP visits, vaccine efficacy, disease burden |  | a vaccine price of D 60 euro result in an ICER of around D 60,000 per QALY |
| Moghadas SM, 2024 |  |  |  |  |
| Li X, 2022 | DSA | The assumed prices for mAb and MI |  | To identify the most influential input parameters, extensive scenario analyses were performed with different assumptions for interventions’ efficacy, duration of protection, disease burden, and accounting for RSV hospitalization-associated recurrent wheeze and asthma |
| Li X, 2023 | DSA | Efficacy; QALY loss; Duration |  |  |
| Rudd M, 2025 |  |  |  |  |
| Gebretekle GB, 2024 | DSA | ①Nirsevimab price per dose; ②Cost, ICU for high-risk infants; ③PVZ price per dose |  | assuming no caregiver costs |
| Liu D, 2021 | DSA | ①Rate of inpatient; ②Rate of death; ③Costs |  |  |
| La EM, 2024 | PSA DSA | ①Annual incidence of RSV-ARI (first event); ②VE against RSV-LRTD; ③Probability of death given RSV-LRTD | The probabilistic sensitivity analysis showed that the adjuvanted RSVPreF3 vaccine was cost-effective in 100.0% of simulations using a willingness-to-pay threshold of $150,000 per QALY gained, in 99.8% of simulations using a willingness-to-pay threshold of $100,000 per QALY gained, and in 91.2% of simulations using a willingness-to-pay threshold of $50,000 per QALY gained (Figure 4). A total of 6.0% of simulations resulted in adjuvanted RSVPreF3 being cost saving (i.e., resulting in improved health outcomes and reduced societal costs) | evaluating cost-effectiveness from the healthcare sector perspective; a 3-year time horizon |
| Hutton DW, 2024 | DSA | GSK, age >65 years: ①Incidence of RSV hospitalizaon; ②VE against hospitalizaon and ED S2;③Vaccine Cost Pfizer, age >65 years: ①VE against hospitalizaon and ED S2; ②Incidence of RSV hospitalizaon; ③Vaccine Cost |  | Scenario analyses with varying RSV incidence and vaccine cost, ICERs, ($/QALY), age ≥ 65 years, 2-year timeframe. |
| Huerta JL, 2025 | PSA DSA | ①Effectiveness of maternal vaccine; ②Incidence of RSV hospitalization; ③Cost of maternal vaccine | The acceptability curves showed that the probability for the maternal vaccine being cost-effective at a WTP of 1–3×GDPpc per QALY gained was 64% to 93% compared to no vaccine | varying discount、vaccine uptake、fatality rate |
| Nourbakhsh S, 2021 |  |  |  |  |
| Nazareno AL, 2025 | PSA DSA | ①Vaccine price; ②Duration of protection; ③Vaccine administration cost | At an assumed vaccine price of $AU 75, 99% of simulations in the PSA were predicted to be cost-effective at the base case WTP threshold. The proportion of simulations predicted to be cost-effective was reduced to about 13% at an assumed vaccine price of $AU 150 and 0% at $AU 250 | Scenario analysis exploring the effect of varying duration of vaccine protection (6 m to 9 m) in newborns and vaccine price ($AU 25 to $AU 300) |
| Koltai M, 2023 | PSA |  | In this case, the ICERs remain below the 2000 (KEN) and 6000 (ZAF) USD per DALY averted thresholds up to dose prices of approximately 75 (KEN) and 125 USD for MV and 100 (KEN) and 150 (ZAF) USD for mAb. |  |
| Hodgson D, 2020 |  |  |  |  |
| Guiñazú G, 2024 | DSA PSA | ①efficacy; ②PPD; ③efficacy duration | Per the reference WTP threshold of 0.9 GDP/capita, both strategies were cost-effective in 100% of the simulated scenarios at baseline PPD. Should the WTP threshold be increased to 1.2 GDP/capita, then a PPD of US$100 would have approximately 80% probability of being cost-effective for both interventions | (i) higher/lower coverage of both interventions; (ii) reduced duration of maternal vaccination protection with increased efficacy: 81.8% for 3 months [13]; (iii) gradual waning for RSV mAb and maternal RSV vaccine protection assuming a gamma curve with an initial maximum efficacy and a gradual reduction in protection reaching zero at 12 months [18]; and, (iv) seasonal dose administration for RSV mAb and maternal RSV vaccine. |
| Zeevat F, 2022 | DSA PSA | ①vaccination cost; ②RSV incidence; ③QALYs lost per hospitalization | If a WTP threshold of €50 000 per QALY is used, the probability of being cost-effective increased to 1. | (1) without discounting QALYs, (2) considering only GP visits and hospitalization incidence rates (excluding cases without any HCP context), (3)targeted vaccination of the high-risk group, (4) vaccination of the ≥75-year-old population, and (5) inclusion of vaccination acquisition costs only (waiving the administration costs). |
| Li X, 2020 | DSA | ①the age-specific RSV hospitalisation probability; ②RSV incidence rate; ③hospital case-fatality ratio |  | price, efficacy, and duration of protection |
| Shoukat A, 2024 | DSA PSA | ①Length of stay in GW without ICU admission; ②Mechanical ventilation days in ICU; ③Proportion of ICU patients using MV | At the WTP of $70,000 per QALY gained, the probability of being cost effective is 54%. | WTP$70000/QALY、vaccination of community-dwelling older adults included either only those aged ≥ 65 years or only those aged ≥ 75 years |
| Baral R, 2020 | DSA | ①intervention efficacy; ②duration of protection; ③anticipated coverage |  | varied efficacy and duration of protection |
| Do LAH, 2023 | DSA PSA | ①efficacy; ②dose price; ③RSV mortality rate | At a WTP threshold of 1-times the national GDP per capita a dose price of less than $15 would have a high probability of being cost-effective (probabilistic clouds are entirely below the WTP threshold line). However, should the WTP threshold be lower (e.g., 0.25 times the national GDP per capita), the dose price would need to be less than $5/dose to have a high probability of being cost-effective. | (1) We assumed a pooled age distribution of RSV disease from six LMICs and assumed that iE could not become negative. A similar method was used for infant mAb. (2) For maternal vaccination we assumed efficacy of 81.8 % against severe RSV disease and 57.1 % against non-severe RSV disease for a three-month period, and zero protection thereafter. This scenario was not applicable to infant mAb. (3) Price reduced to $15 per dose. (4) Price reduced to $5 per dose. |
| Laufer RS, 2023 | DSA PSA | ①infant vaccine effectiveness; ②vaccine price; ③RSV incidence | Probability each RSV LRTI prevention strategy is optimal given a product price per dose and willingness-to-pay of $891, equal to the per capita GDP of Mali. | Maternal vaccine coverage was 89.1% |
| Pouwels KB, 2016 | DSA PSA | ①vaccine effectiveness; ②RSV incidence; ③vaccine price | At a threshold of 61,821 TL, 90%, 51% and 44% of Monte Carlo samples are considered cost-effective for infant vaccination only, pregnant women vaccination only and combination of both strategies, respectively |  |
| Hodgson D, 2024 | DSA | coverage rates |  |  |
| Tuite AR, 2024 | DSA PSA |  | Probabilistic sensitivity analysis identified some uncertainty about the optimal strategy around the $50 000 per QALY thresh old, with the risk based strategy most likely to be cost-effective shifting from age 80 years to age 70 years for both vaccines near this threshold (Appendix 1, Supplementary Figure 2). Risk based vaccination of people aged 70 years and older had the largest probability of being cost-effective from $50 000 up to a threshold of $80 000 per QALY. Beyond $80 000 per QALY, there was a less clear difference between the age 60 years and age 70 years risk based strategies. | vaccine protection extends through a third RSV season |
| Meijboom MJ, 2013 | DSA | ①the RSV-associated mortality rate; ②the life expectancy of HR elderly; ③baseline quality of life of elderly |  |  |
| Gessner BD, 2000 | PSA |  | For the best and worst case scenarios, the vaccine effectiveness was held constant at 80% against hospitalization and death and 50% against outpatient illness, while the cost was held constant at US$30 |  |
